# Clone and Function Verification of the *OPR* gene in *Brassica napus* Related to Linoleic Acid Synthesis

**DOI:** 10.1186/s12870-022-03549-1

**Published:** 2022-04-12

**Authors:** Min Tan, Juan Niu, Duo Zi Peng, Qian Cheng, Ming Bao Luan, Zhen Qian Zhang

**Affiliations:** 1 College of Agronomy, Hunan Agricultural University, Changsha, 410128 Hunan China; 2 Institute of Bast Fiber Crops, Chinese Academy of Agricultural Sciences, Changsha, 410205 Hunan China

**Keywords:** Rapeseed, *OPR* gene, Linoleic acid, RNAi, Overexpression

## Abstract

**Background:**

Fatty acid composition and content affect rapeseed oil quality. Fatty acid synthesis-related genes in rapeseed have been studied globally by researchers. Nevertheless, rapeseed oil is mainly composed of seven different fatty acids (FA), and each fatty acid was regulated by different genes. Furthermore, different FA affect each other, which needs continuous and in-depth research to obtain more clear results in *Brassica napus*.

**Results:**

In this paper, broad-scale miRNA expression profiles were constructed and 21 differentially expressed miRNAs were detected. GO enrichment analysis showed that most up-regulated proteins were involved in transcription factor activity and catalytic activity. KEGG pathway enrichment analysis indicated that 20 pathways involving 36 target genes were enriched, of which the *bna00592* pathway may be involved in fatty acid metabolism. The results were verified using a quantitative real-time PCR (RT-qPCR) analysis, we found that the target gene of *bna-miR156b* > *c* > *g* was the *OPR* (12-oxo-phytodienoic acid reductase). Four copies of *OPR* gene were found, and the over-expression vectors (pCAMBIA1300-35 s-*OPR* and pCAMBIA1300-RNAi-*OPR*) were constructed to verify their functions. In T_1_ and T_2_ generation, the content of linoleic acid (LA) increased significantly in OE but deceased in OPRi.

**Conclusions:**

This is the first study to provide four copies of the *OPR* gene that regulates LA metabolism, can be used for the molecular mechanism of LA and optimizing fatty acid profiles in oilseed for breeding programs.

**Supplementary Information:**

The online version contains supplementary material available at 10.1186/s12870-022-03549-1.

## Background

*Brassica campestris* L*.* (rapeseed) is one of the most important oil crops in the world [1, 2]. The quality of rapeseed oil mainly depends on its fatty acid composition, especially the proportion of three main unsaturated FA: oleic acid (OA, C_18: 1_), LA (C_18: 2_), and linolenic acid (C_18: 3_) [3]. Many studies have shown that rapeseed oil with a high unsaturated fatty acid content will have better health effects and can prevent the occurrence of cardiovascular diseases [4]. LA can prevent or reduce the incidence of cardiovascular diseases and being used for the prevention and treatment of hypertension, hyperlipidemia, angina pectoris, coronary heart disease, atherosclerosis, and senile obesity [5, 6]. LA could be hydrogenated to produceα-linolenic acid (ALA) andγ-linolenic acid (GLA) [7].

miRNAs are short noncoding regulatory RNAs that regulate gene expression via post-transcriptional repression [8, 9]. In plants, miRNAs are involved in various biological processes, including the regulation of plant development [10], architecture formation [11], photosynthesis [9], tolerance to biotic and abiotic stresses [12–15]. In recent years, several new miRNAs have been found in oilseed rape [16–18]. The process of seed development is a period that starts at embryo development and ends when dry seeds are mature; it is a key stage that affects the seed size, oil production, protein content, and antinutritional accumulation of rapeseed. Furthermore, gene expression changes during seed development [19–21]. Recently, miRNAs and their target transcripts involved in fatty acid and lipid metabolism have been studied in developing seed of *Brassica napus*, which is considered the third largest oil crop worldwide [22, 23]. The generation of acetyl-CoA and carbon chain desaturase were also observed; nevertheless, the total number of known miRNAs and their functions in *Brassica napus* are still unknown [23].

The development of molecular biology technology has greatly promoted the research on LA breeding in rapeseed. (I) In terms of mining the function of genes, a study showed significant correlation between *FAD3* and LA content [24], while another study revealed that the overexpression of *SsDGAT1* significantly affected LA content (about 16%) [25]. *McD6DES* generates a double bond at the carboxyl end of LA, which reduces the LA content [26]; *BJULFY* increases LA content (approximately 5%) [27]. The single nucleotide mutation of *FAD3* exon from G to A was screened from the rapeseed “Alboglabra”, which treated with an Ethyl Methyl Sulfone (EMS) solution, and the mutant materials with high LA content and low alanine content (about 2.0%) were obtained [28]. (II) In terms of screening the genes, a genome wide association study (GWAS) discovered 53 and 24 SNP related to LA in 2013Cq and 2014Cq rapeseed materials, respectively [29]; when combined with RT-qPCR, 95 candidate genes were found to be highly related to LA and other fatty acid metabolism [30]. (III) In terms of locating the genes, *FAD2* gene on chromosome A05 was found to be associated with LA content, and minor gene regulating LA content was found on chromosome A09 [31]; twenty QTLs related to LA were detected using two spring rapeseed varieties, “Polo” and “Topas”, and subsequently distributed in seven linkage groups: A01, A02, A03, A05, C01, C03, and C09 [32]. In fact, many studies indicate that OA metabolism depend on *FAD1* or *FAD2* activity [33, 34], and the have present the first evidence of Δ7 desaturation via the *FADS1* gene product by Hui et al.[35] LA are used as precursors of ultra-long chain polyunsaturated FA, such as α-linolenic acid (ALA) and γ-linolenic acid (GLA), which are hydrogenated by desaturation enzymes [36]. Correlations between oleic versus linoleic and oleic versus linolenic were negative and highly significant, and correlations between linoleic versus linolenic were of lower magnitude by Kondra et al. [37]. According to Belo et al. [38], the impact of presenting an interesting study focusing on environmental effects on oil quality of oil crops, who showed that the differential effects of climate conditions had an effect on fatty acid composition.

miRNA technology has been adopted to find several miRNAs in enzymes used for carbon chain desaturation, while being used in studies on fatty acid and lipid metabolism in rapeseed [22]. Employing this technology can be conducive to the breeding of rapeseed [23]. Therefore, we sequenced rapeseed material using miRNA technology. Then, we excavated four copies of *OPR* genes. The four copies of gene overexpression and RNAi vectors were transferred into *A. thaliana*. This is the first study to report that all copies of *OPR* gene could directly regulate the synthesis of LA. These findings will enhance the understanding of LA metabolism in rapeseed.

**Results**

### Overview of sRNA sequencing results

**Fig. 1:**
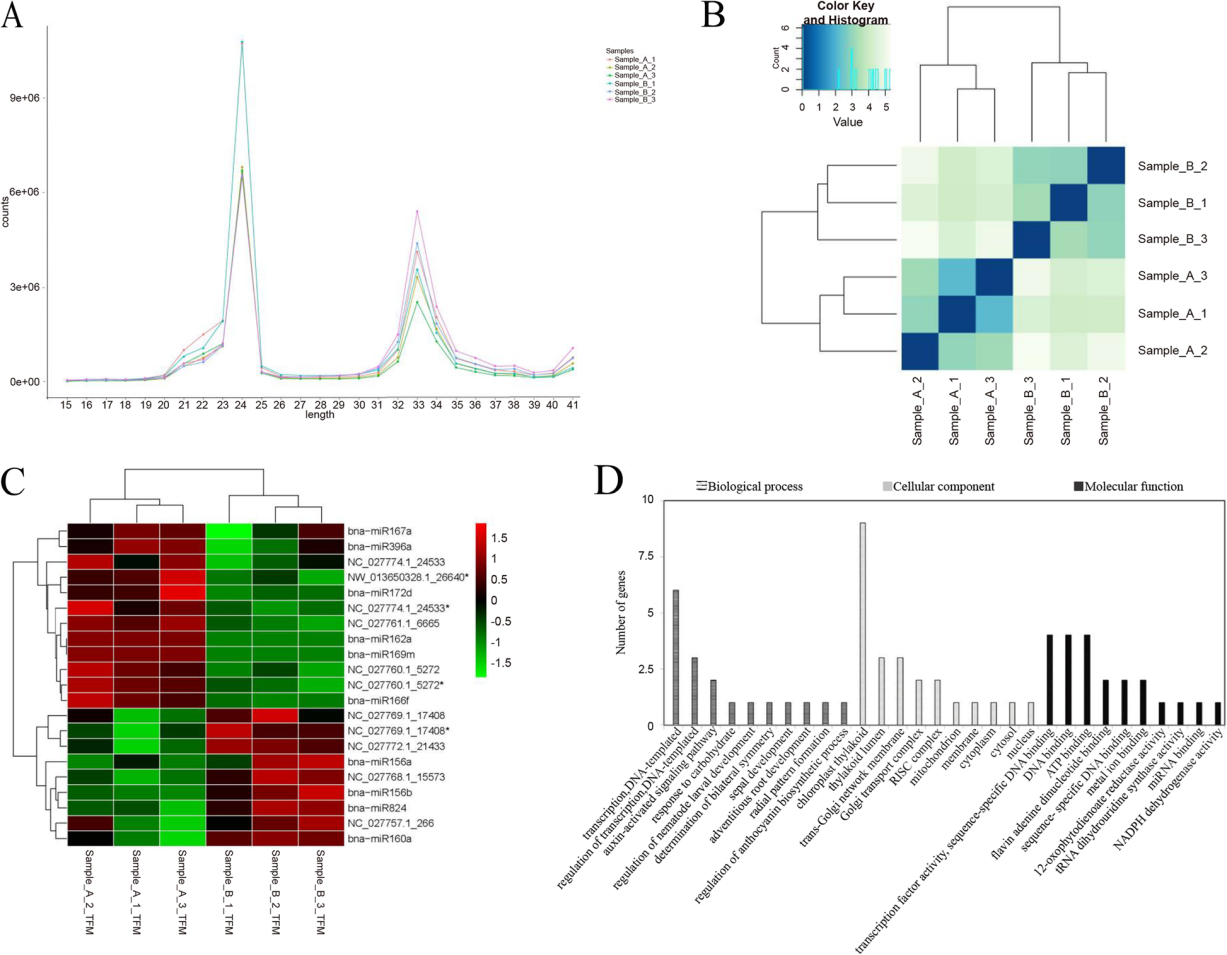
Sample size, sample-to-sample cluster and miRNAs cluster, GO enrichment analysis (A) Size distribution of the clean reads; (B) Results of sample-to-sample cluster analysis, Sample-A: Low oleic acid rapeseed materials; Sample-B: High oleic acid rapeseed materials; (C) Cluster distribution diagram of the different miRNAs; (D) GO enrichment analysis TOP10

We have already upload data and ensure the deposited data is made public (NCBI): https://www.ncbi.nlm.nih.gov/sra/PRJNA760803, accession number: PRJNA760803. A total of 22,744,964 (LOAR: low OA rapeseed materials, A) and 26,060,122 (HOAR: high OA rapeseed materials, B) raw reads were generated from the sequencing machine. After removing the adaptor sequences, filtering out low quality tags, and cleaning up sequences derived from adaptor ligation, 20,912,776 (A) and 23,710,938 (B) clean reads were obtained. Consequently, the bioinformatic analysis of these clean reads were carried out (Table 1). The size distribution patterns of the original and unique reads were displayed in Fig. 1A. Small RNAs (24 nt) were the most abundant in all the samples. In addition, the clean reads exhibited 87.45% (A) and 88.26% (B) homology with the reference genome sequences. The sRNA sequencing results were of high quality and reliable and can be used for further functional analysis.

**Table 1:** Statistics of sRNA sequences

		A1	A2	A3	A mean value	B1	B2	B3	B mean value
raw reads		3E + 07	2E + 07	1.8E + 07	22,744,964	2.6E + 07	2.4E + 07	2.8E + 07	26,060,122
clean reads		2.7E + 07	1.9E + 07	1.7E + 07	20,912,776	2.5E + 07	2.1E + 07	2.5E + 07	23,710,938
uniq reads		4,723,178	3,183,059	3,150,216	3,685,484	4,291,822	3,249,172	3,403,565	3,648,186
reference genome matches		87.39%	87.52%	87.44%	87.45%	88.61%	88.19%	87.99%	88.26%
Annotation type	rRNA	2.84%	2.93%	2.64%	2.80%	3.20%	4.44%	4.40%	4.01%
	tRNA	32.77%	38.46%	32.80%	34.68%	29.42%	43.11%	47.02%	39.85%
	snRNA	0.63%	0.66%	0.57%	0.62%	0.51%	0.70%	0.72%	0.64%
	Cis reg	0.09%	0.09%	0.09%	0.09%	0.10%	0.10%	0.10%	0.10%
	others	0.28%	0.27%	0.26%	0.27%	0.30%	0.34%	0.34%	0.33%
	gene	0.40%	0.40%	0.37%	0.39%	0.34%	0.49%	0.52%	0.45%
	repeat	2.23%	2.52%	2.03%	2.26%	2.55%	3.78%	3.97%	3.43%
Aligned reads		0.60%	0.45%	0.51%	0.52%	0.64%	0.42%	0.43%	0.50%
Aligned know miRNA		39	38	39	38.67	36	35	37	36
Note: A: Low oleic acid rapeseed materials; B: High oleic acid rapeseed materials

### Differentially expressed miRNAs identification and their function analysis

The clustering analysis method was used to investigate the similarity between samples by calculating the differential miRNA distance between LOAR (A) and HOAR (B) (Fig. 1B). As shown, three samples of high or low OA materials were found in a cluster, suggesting that these miRNAs might have similar biological functions.

After applying a *P*-value < 0.05 and an absolute value of log2 (treatment/control: LOAR/HOAR) greater than 1.5 to identify differentially expressed miRNAs, 21 differentially expressed miRNAs (8.39% of the total) were detected (Table 2, Supplemental Table 2); their frequencies were calculated using TPM (Table 3). Among them, nine genes (42.86%) were up-regulated and 12 (57.14%) were down-regulated in HOAR materials (Fig. 1C). Briefly, 21 miRNAs (including bna-miR824, bna-miR396a, bna-miR172d, bna-miR169m, bna-miR167a > b, bna-miR166f, bna-miR162a, bna-miR160a > b > c > d, bna-miR156b > c > g, bna-miR156a, NW_013650328.1_26640*, NC_027774.1_24533*, NC_027774.1_24533, NC_027772.1_21433, NC_027769.1_17408*, NC_027769.1_17408, NC_027768.1_15573, NC_027761.1_6665, NC_027760.1_5272*, NC_027760.1_5272, NC_027757.1_266) and 358 target genes were obtained. Target finder software was used to analyze the target genes of differential miRNAs, refer to Fahlgren and Carrington (2010) [62]. In a word, 21 miRNAs were found to target 358 genes. Subsequently, according to *p*-value < 0.05, 133 putative target genes were obtained by screening significantly enriched GO and KEGG pathways from 358 target genes.

**Table 2:** Primers for real time PCR analysis of miRNA

miRNA name	Primer Sequence
NC_027757.1_266	TGCCTGGCTCCCTGTATACCA
NC_027760.1_5272	TTGGAGGACTGGTGATGAAAAC
NC_027760.1_5272*	TTGTAACAGCTTTTAGTCCTCTT
NC_027761.1_6665	ATACTTAGAGCCTTATTACGCCT
NC_027768.1_15573	ACCTTGTTTTGGTCGGACGAG
NC_027769.1_17408	CAGTTTTGTAAGTTCTGTCCAG
NC_027769.1_17408*	GGTTGTTACTTATACGGCTATA
NC_027772.1_21433	CACAATCGCCCTTGAAGCTG
NC_027774.1_24533	CGAGTGTGAAGAATGCGGCG
NC_027774.1_24533*	GATCCTTCTCGAGAAACTGGC
NW_013650328.1_26640*	CTTTGCCTATCGTTTGGAAAAG
bna-miR156a	TGACAGAAGAGAGTGAGCACA
bna-miR156b > bna-miR156c > bna-miR156g	TTGACAGAAGATAGAGAGCAC
bna-miR160a > bna-miR160b > bna-miR160c > bna-miR160d	TGCCTGGCTCCCTGTATGC
bna-miR162a	TCGATAAACCTGTGCATCCAG
bna-miR166f	TCGGACCAGGCTTCATCCC
bna-miR167a > bna-miR167b	TGAAGCTGCCAGCATGATCTAA
bna-miR169m	TGAGCCAAAGATGACTTGCCG
bna-miR172d	AGAATCTTGATGATGCTGCAG
bna-miR396a	TTCCACAGCTTTCTTGAACTT
bna-miR824	TAGACCATTTGTGAGAAGGGA

**Table 3:** TPM values of 21 differential miRNAs in Samples

miRNA_id	A1 TPM	A2 TPM	A3 TPM	A TPM mean values	B1_TPM	B1_TPM	B1_TPM	B TPM mean values
NC_027757.1_266	39.78041213	89.29803626	28.94628294	52.67491044	66.49508328	103.5718478	135.4255601	101.8308304
NC_027760.1_5272	322.2213382	430.2541747	274.9896879	342.4884003	132.9901666	165.7149565	117.7613566	138.8221599
NC_027760.1_5272*	879.147108	1095.930445	817.7324929	930.9366819	434.173779	393.5730216	312.067595	379.9381319
NC_027761.1_6665	1996.976689	2256.804916	2330.175776	2194.65246	1384.662322	1277.386123	1165.83743	1275.961959
NC_027768.1_15573	1272.973188	1493.712606	1389.421581	1385.369125	1721.049214	2092.151325	1937.174316	1916.791619
NC_027769.1_17408	2784.628849	3547.56744	3046.596279	3126.264189	3809.777124	4439.779875	3450.407749	3899.988249
NC_027769.1_17408*	83.53886546	113.6520461	115.7851317	104.3253478	195.5737743	151.9053768	153.0897636	166.8563049
NC_027772.1_21433	1241.148858	1542.420626	1425.604435	1403.057973	1975.295121	1898.817209	1789.97262	1888.028317
NC_027774.1_24533	198.9020606	332.8381351	296.6994001	276.1465319	121.2557401	158.8101666	194.3062384	158.1240484
NC_027774.1_24533*	787.6521601	1152.756468	926.2810539	955.5632273	621.9246024	552.3831882	594.6948509	589.6675472
NW_013650328.1_26640*	27,770.7057	26,415.98272	39,605.75163	31,264.14669	16,392.99377	19,395.5547	14,502.31107	16,763.61984
bna-miR156a	1917.415864	1574.892639	1599.282132	1697.196879	1795.367248	2768.820731	2926.369712	2496.852564
bna-miR156b > c > g	1149.65391	1250.172508	1121.668464	1173.831627	1799.278724	2271.675862	2861.600966	2310.85185
bna-miR160a > b > c > d	449.518657	552.0242241	383.5382489	461.69371	645.3934553	704.288565	677.1278005	675.6032736
bna-miR162a	83.53886546	138.006056	72.36570734	97.97020961	0	0	0	0
bna-miR166f	76,732.43695	89,371.09828	71,996.64223	79,366.72582	52,182.99447	49,652.34383	50,655.04755	50,830.12862
bna-miR167a > b	3751.292863	3287.791335	3661.704791	3566.929663	2256.921356	2969.059637	3526.95263	2917.644541
bna-miR169m	15.91216485	24.35400989	21.7097122	20.65862898	0	0	0	0
bna-miR172d	473.3869043	454.6081846	752.6033563	560.1994817	269.8918086	296.9059637	288.5153237	285.1043653
bna-miR396a	751.8497892	576.378234	723.6570734	683.9616989	340.2983674	428.0969709	577.0306474	448.4753285
bna-miR824	700.1352534	681.9122769	549.9793758	644.0089687	907.4623129	1180.719065	1118.732888	1068.971422

The target genes were then subjected to GO functional and KEGG Pathway analyses. In many cases, multiple terms were assigned to the same miRNA. Thus, 133 putative target genes were associated with 21 differentially expressed miRNAs and distributed into the following subcategories: 62 “Biological process”, 32 “Cellular component”, and 39 “Molecular function” (Fig. 1D). Under “Biological process”, most of the target genes were related to “transcription”, and “regulation of transcription”. Within the “Cellular component” category, “nucleus” and “cytosol” were observed as much as “cytoplasm”. Among genes in the “Molecular function” category, most potential functions were related to “transcription factor activity”, “DNA binding”, and “Catalytic activity”. The distribution of target genes indicated that rapeseed underwent active metabolization.

**Fig. 2:**
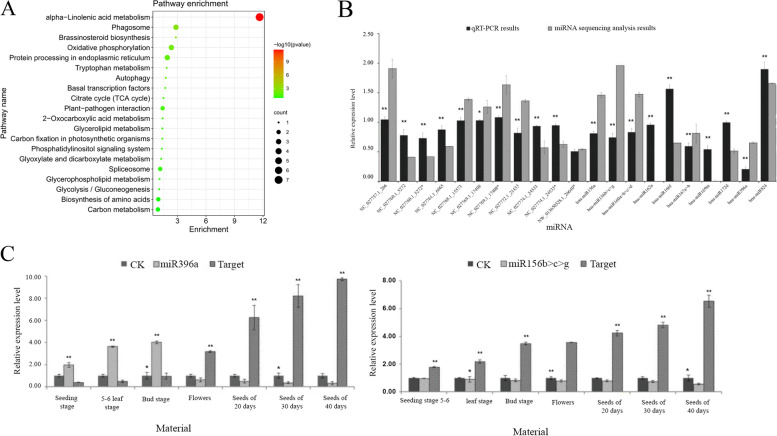
KEGG enrichments, RT-qPCR results of miRNAs and the expressions of miRNAs and their target genes (A) Top 20 KEGG enrichments; (B) The RT-qPCR results of 21 differential miRNAs. The results of low oleic acid rapeseed materials were used as control. (* and ** signify the difference level of P < 0.05 and P < 0.01, respectively); (C) The expressions of miRNAs (miR396a, miR1566b > c > g) and their target genes. The expression of miRNA and its target gene were used as controls

The KEGG pathway enrichment analysis indicated that 20 pathways involving 36 target genes were enriched, including “alpha-Linolenic acid metabolism” (7 target genes), “Phagosome” (3 target genes), “Oxidative phosphorylation” (3 target genes), “Oxidative phosphorylation” (3 target genes), and “Protein processing in endoplasmic reticulum” (3 target genes). The fifteen target genes may be related to fatty acid metabolism: “alpha-linolenic acid metabolism” (7 genes), “Oxidative phosphorylation” (3 genes), “Carbon metabolism” (2 genes), “Citrate cycle (TCA cycle)” (1 gene), “Glycerolipid metabolism” (1 gene), and “Glycolysis / Gluconeogenesis” (1 gene). The top 20 KEGG enrichments (Fig. 2A) show that α-Linolenic acid metabolism is the most significant, suggesting that *bna00592* KEGG pathway may be involved in fatty acid metabolism in rapeseed (Fig. 2B).

### Expression pattern of *bna-miR156b* > *c* > *g* gene was detected by RT-qPCR

To confirm the results of the miRNA sequence analysis, 21 annotated differentially expressed miRNAs were compared to the *Brassica napus* genome using BLAST [39] (Fig. 2B).

Most expression trends of the RT-qPCR analysis results agreed with the miRNA sequencing data (NC_027757.1_266, NC_027760.1_5272, NC_027760.1_5272*, NC_027761.1_6665, NC_027768.1_15573, NC_027769.1_17408, NC_027769.1_17408*, NC_027774.1_24533, NC_027774.1_24533*, NW_013650328.1_26640*, *bna-miR162a*, *bna-miR167a* > *b*, *bna-miR169m*, *bna-miR172d*, *bna-miR396a*, *bna-miR824*.), In addition, among the 13 miRNAs with significant difference, 9 had target genes (NC_027760.1_5272, NW_013650328.1_26640*, *bna-miR156b* > *c* > *g*, *bna-miR166f*, *bna-miR169m*, *bna-miR396a*, *bna-miR824*, *bna-miR156a,* and *bna-miR160a* > *b* > *c* > *d*), which may be the novel miRNAs related to FA.

Moreover, the expressions of fatty acid metabolism related to differential miRNAs, such as *bna- miR396a*, *bna-miR156b* > *c* > *g,* and their target genes, were studied in different developmental stages (Fig. 2C). The *bna-miR396a* has opposite expression pattern with its target gene, at first, the *bna-miR396* had up-regulated expression, until the bud stage reached the peak, and the expression decreased with the growth stage; *bna-miR156b* > *c* > *g* had an opposite expression pattern with its target gene, it had down-regulated expression in the whole growth stages, and the expression decreased with the growth stages. Differentially expressed miRNAs and their target genes were related to fatty acid metabolism in *bna-mi156b* > *c* > *g* at different developmental stages (Fig. 1C). In contrast to the expression pattern of *bna-miR156b* > *c* > *g* and its target gene, the expression level was down-regulated throughout the whole growth stage of the plant, and the expression level gradually decreased with the growth process of rapeseed.

### Cloning of *OPR* genes in rapeseed and bioinformatic analysis

**Fig. 3:**
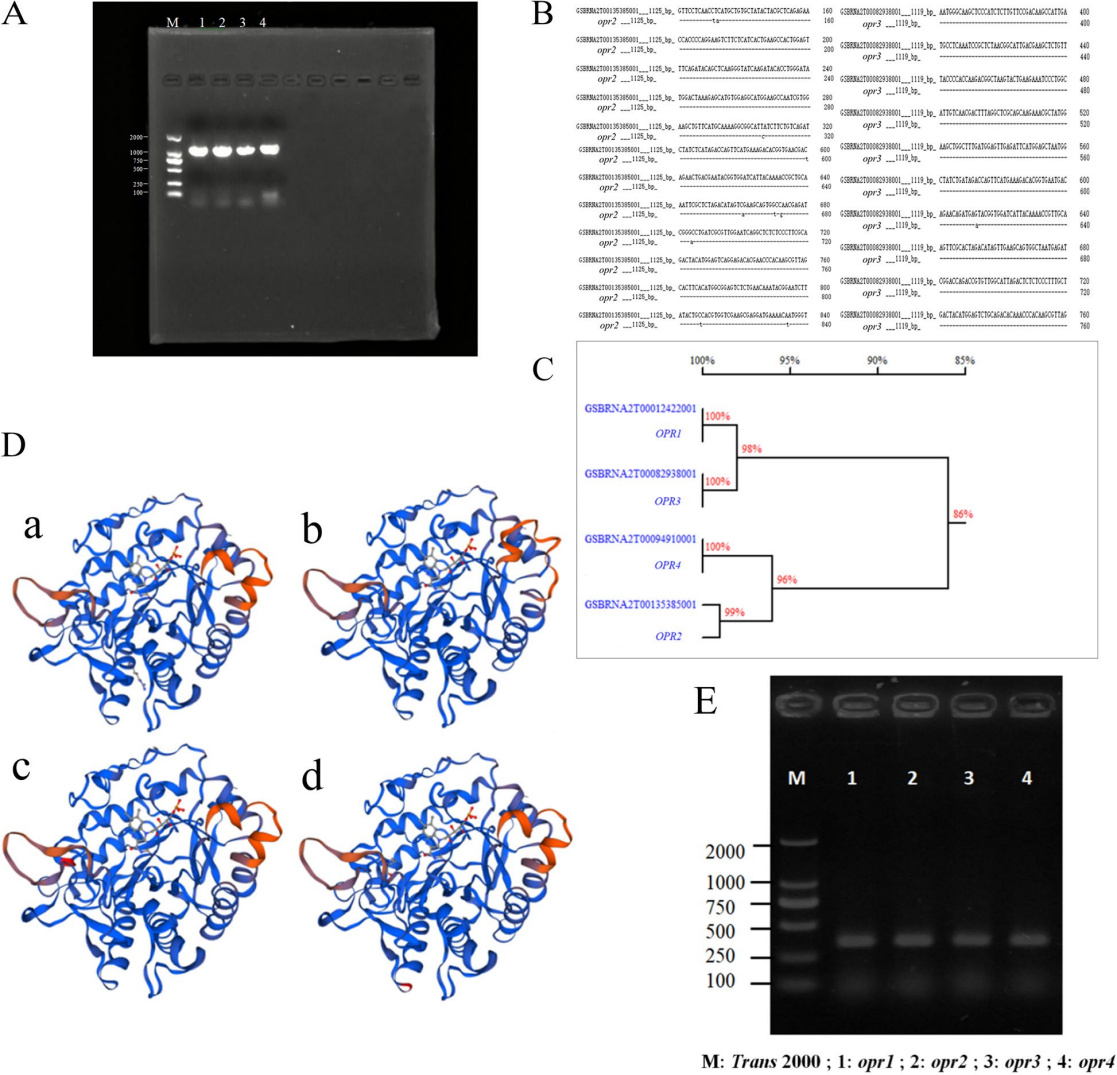
*OPR* genes PCR amplification, sequence and homology and the tertiary structure of protein analysis, RNAi fragment PCR amplification (A) Target gene PCR amplification (M: *Trans* 2000 bp; 1: *OPR1*; 2: *OPR2*; 3: *OPR3* 4: *OPR4*); (B) Sequence alignment with the sequence published in Brassica Database; (C) Sequence homology analysis with Brassica Database; (D) Tertiary structure of protein (A. *OPR1*; B. *OPR2*; C. *OPR3*; D. *OPR4*); (E) Objective Gene RNAi fragment PCR electrophoresis map

Target gene: *bna-miR156b* > *c* > *g* was cloned by miRNA sequencing, and four copies were detected: *GSBRNA2T00012422001*, *GSBRNA2T00135385001*, *GSBRNA2T00082938001*, and *GSBRNA2T00094910001*, which were named *OPR1*, *OPR2*, *OPR3*, and *OPR4*, respectively. Both *OPR1* and *OPR3* were 1119 bp, *OPR2* and *OPR4* were 1125 bp and 1122 bp, respectively (Fig. 3A).

DNAMAN 7.0 software was used to compare the cloned target sequence with the rapeseed sequence published on the *B.napus* Genome Browser website. Different base position (Fig. 3B), homology was more than 99% with the published sequences (Fig. 3C). *OPR1*, *OPR3*, *OPR4* had no base difference with the published sequence, there were 10 base differences between *OPR2* and published sequence and the homology was 99.11%. Preliminary identification of *OPR2* and *OPR3* were located in A genome and *OPR4* and *OPR1* in C genome was conducted.

The number of four copies of *OPR* gene amino acids ranged from 372 to 374 bp, with a molecular weight of about 41 ku; The encoded amino acids were acidic (< 7), unstable (< 40), exhibited a fat coefficient of about 75, and belonged to fat-soluble proteins. Predicted subcellular localization of proteins were encoded by different copies of *OPR* genes and we found that these four proteins were located in the cytoplasm. We found that the four copies were all extracellular proteins without a transmembrane structure. Predicted protein secondary structures were summarized in Table 4.

**Table 4:** Primary and secondary structure analysis of each copy

structure	Analysis	*OPR*1	*OPR*2	*OPR*3	*OPR*4
Physical and chemical properties of primary structures	Number of amino acids	372	374	372	373
	Molecular weight	41.4	41.57	41.32	41.28
	Theoretical pI	5.55	6.01	5.88	6.22
	Total number of positively charged residues(Arg + Lys)	46	44	43	42
	Total number of negatively charged residues(Asp + Glu)	37	38	37	38
	Formula	C_1844_H_2838_N_508_O_550_S_15_	C_1844_H_2863_N_511_O_554_S_16_	C_1843_H_2839_N_509_O_546_S_15_	C_1835_H_2843_N_509_O_546_S_16_
	Instability index	38.82	35.76	39.65	38.57
	Aliphatic index	75.27	75.64	75.27	75.6
	Grand average of hydropathicity (GRAVY)	-0.355	-0.359	-0.343	-0.325
Secondary structure	Alpha helix (Hh)	31.18	31.55	31.18	30.56
	Beta bridge (Bb)	0	0	0	0
	Extended strand (Ee)	12.63	12.03	12.63	12.87
	Beta turn (Tt)	6.72	6.42	7.26	8.04
	Random coil (Cc)	49.46	50	48.92	48.53

The predicted tertiary structure model of the protein shows that the tertiary structures of *OPR1*, *OPR2*, *OPR3,* and *OPR4* were all adapted to the 12-O-plant dienoate reductase model (integrated with the crystal structure of *At1g76680* protein in *A. thaliana*), but their conformations were slightly different (Fig. 3D). It has been reported that the protein structure *A. thaliana* of *At1g76680* is similar to that of yeast ScOYE1 [40].

### Vector construction and transformation of *A. thaliana*.

**Fig. 4:**
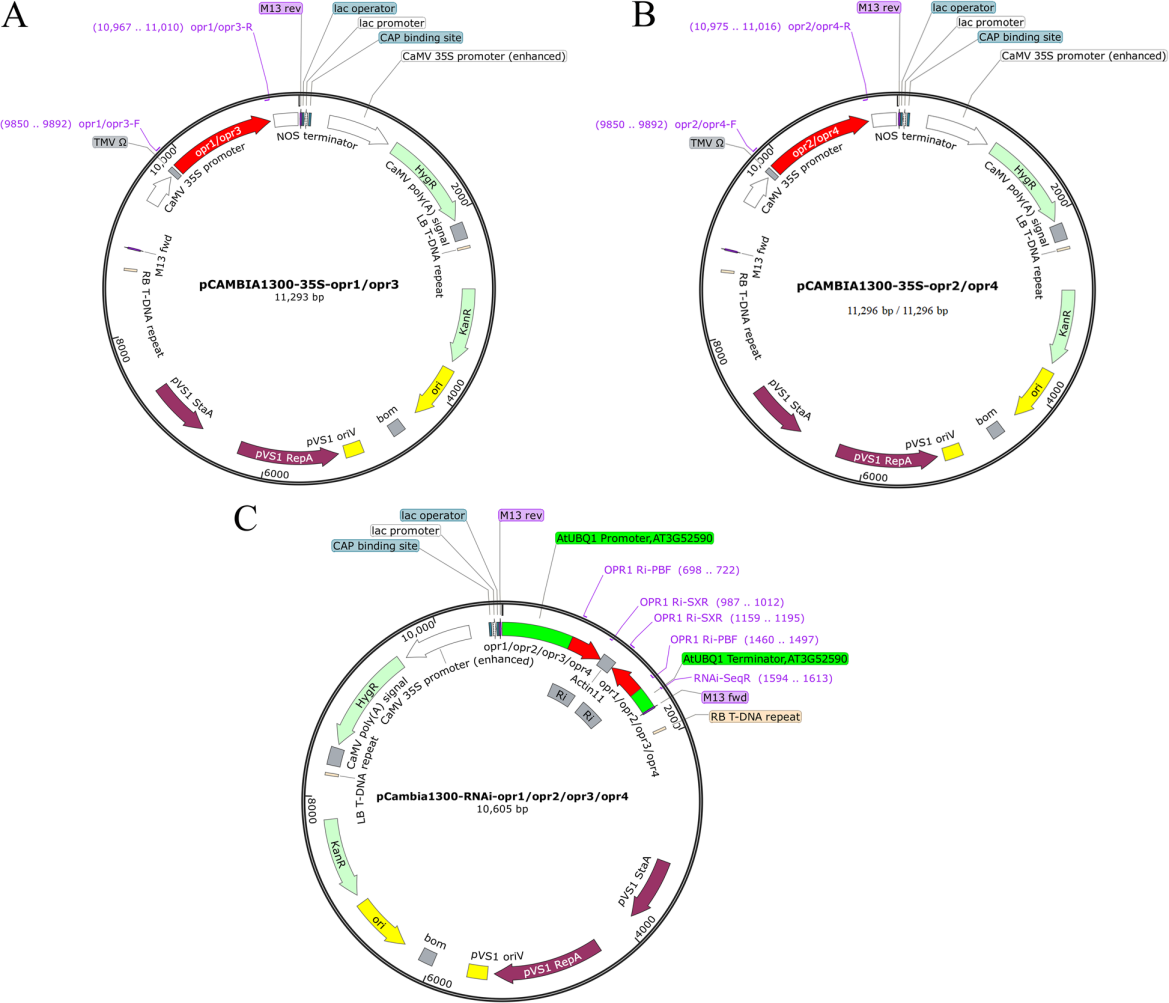
*OPR* genes 35 s and RNAi recombinant vector map (A) and (B) pCAMBIA1300-35 s-*OPR1*/*OPR2*/*OPR3*/*OPR4* recombinant vector; (C) pCAMBIA1300-RNAi-*OPR1*/*OPR2*/*OPR3*/*OPR4* recombinant vector

Using the synthesized cDNA as template, four target gene specific fragments were amplified by PCR with high fidelity, and the RNAi fragments with the same length were obtained (Fig. 3E). Then, the target genes were recombined with the overexpression vector pCAMBIA1300-35 s, positive strains were screened. After sequencing, the overexpression vectors pCAMBIA1300-35 s-*OPR1* (OPR1-OE), pCAMBIA1300-35 s-*OPR2* (OPR2-OE), pCAMBIA1300-35 s-*OPR3* (OPR3-OE)*,* and pCAMBIA1300-35 s-*OPR4* (OPR4-OE) were obtained (Fig. 4A-B).

The RNAi fragment was recombined with the RNAi vector pCAMBIA1300-RNAi and the positive strains were screened. After sequencing, the RNAi vectors pCAMBIA1300-RNAi-*OPR1* (OPR1i), pCAMBIA1300-RNAi-*OPR2* (OPR2i), pCAMBIA1300-RNAi-*OPR3* (OPR3i)*,* and pCAMBIA1300-RNAi-*OPR4* (OPR4i) were obtained (Fig. 4C).

**Fig. 5:**
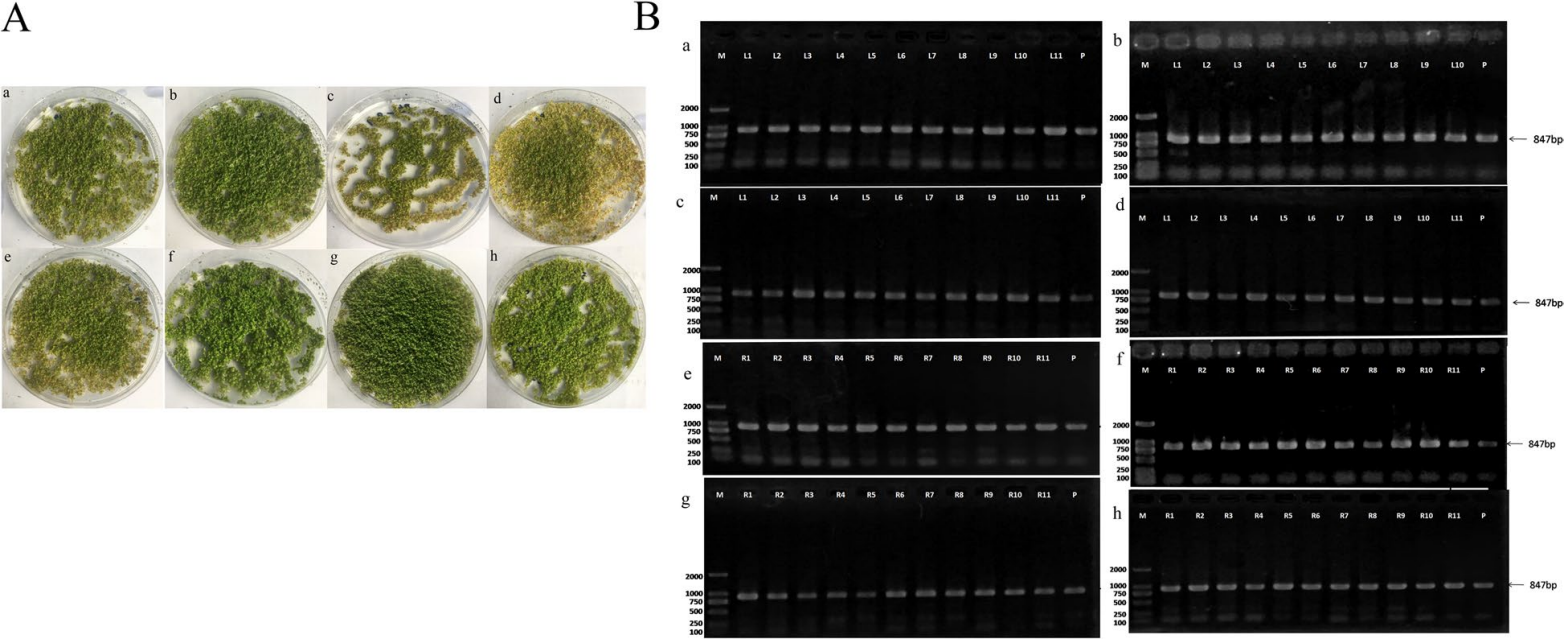
Screening and identification of transgenic *A. thaliana*: (A) Hygromycin screening of transgenic *A. thaliana*. a/c/e/g: *OPR1*/*2*/*3*/*4*-OE; b/d/f/h: *OPR1*/*2*/*3*/*4*-OE; (B) Identification of hygromycin in transgenic *A. thaliana* (M: DL 2000 bp; P: pCAMBIA1300; a/c/e/g: pCAMBIA1300-35 s-*OPR1*/*2*/*3*/*4*; b/d/f/h: pCAMBIA1300-RNAi-*OPR1*/*2*/*3*/*4*)

The recombinant vector was transformed into *A. thaliana* and the obtained *A. thaliana* was detected. The T_1_ transgenic *A. thaliana* seeds were screened using hygromycin (Fig. 5A) and the results of hygromycin primer identification (Fig. 5B) showed that each copy of *A. thaliana OPR* had been successfully transformed, and the target plasmid T-DNA had been inserted into the genome of *A. thaliana*.

### Analysis of fatty acid composition and fatty acid content

The transformation methods in *A. thaliana*, reference Clough and Bent (1998) [41]. fatty acid composition was detected by gas chromatography [42]. we obtained fatty acid composition results of the T_1_ and T_2_ generations. Fatty acid composition of *A. thaliana* T-DNA insertion lines in Table 5, and transformation materials fatty acid composition in the contrast T_1_/T_2_. The contents of OA and stearic acid in OPR1i were significantly increased and the LA content decreased significantly; OPR1-OE will lead to a significant increase in palmitic acid and LA content; OPR2i significantly increased stearic acid content and decreased LA content; and OPR2-OE increased LA content.

**Table 5:** Analysis of fatty acid composition of T1 and T2 seeds

Strain			Saturated fatty acids (%)			Unsaturated fatty acid (%)					
			Palmitic acid	Stearic acid	Total	Oleic acid	Linoleic acid	Linolenic acid	Arachidonic acid	Erucic acid	Total
T_1_	Contrast T_1_		7.851 ± 0.37	2.947 ± 0.14	10.798	15.145 ± 3.51	28.204 ± 2.31	19.115 ± 1.53	19.925 ± 2.05	0 ± 0.06	82.389
	*OPR1*	OPR1i	6.8309 ± 0.277	3.3757 ± 0.221	10.2066	22.5651 ± 2.401	25.1403 ± 1.838	14.231 ± 1.057	18.7597 ± 1.975	1.2141 ± 0.535	81.9102
		Increase or decrease	-12.99%^**^	14.55%^**^	-5.48%^*^	48.99%^**^	-10.86%^**^	-25.55%^**^	-5.85%^*^	-	-0.58%
		OPR1-OE	9.5163 ± 1.048	1.3951 ± 0.438	10.9114	20.5827 ± 1.609	32.2019 ± 1.666	19.79963 ± 7.508	18.3739 ± 3.331	0.2149 ± 0.257	91.17303
		Increase or decrease	21.21%^**^	-52.66%^**^	1.05%	35.90%^**^	14.17%^**^	3.58%^*^	-7.78%^*^	-	10.66%^**^
	*OPR2*	OPR2i	7.2108 ± 0.384	3.2877 ± 0.183	10.4985	16.8825 ± 1.533	26.7364 ± 3.095	17.4696 ± 1.08	19.9638 ± 0.183	0.0473 ± 0.100	81.0996
		Increase or decrease	-8.15%^*^	11.56%^**^	-2.77%^*^	11.47%^**^	-5.20%^*^	-8.61%^*^	0.19%	-	-1.57%
		OPR2-OE	9.1207 ± 0.584	1.1043 ± 0.523	10.225	21.4321 ± 3.32	31.029 ± 1.838	17.0149 ± 1.747	18.1661 ± 2.313	0.3125 ± 0.323	87.9546
		Increase or decrease	16.17%^**^	-62.53%^**^	-5.31%^*^	41.51%^**^	10.02%^*^	-10.99%^*^	-8.83%^*^	-	6.76%^*^
	*OPR3*	OPR3i	7.3383 ± 0.438	2.8731 ± 1.018	10.2114	18.318 ± 2.432	27.7582 ± 1.057	17.5874 ± 1.266	19.3568 ± 0.831	0.1795 ± 0.384	83.1999
		Increase or decrease	-6.53%^*^	-2.51%	-5.43%	20.95%^**^	-1.58%	-7.99%^*^	-2.85%	-	0.98%
		OPR3-OE	10.2309 ± 1.924	1.351 ± 0.566	11.5819	22.9638 ± 1.382	32.2931 ± 3.819	18.9167 ± 2.314	14.2964 ± 1.938	0.2542 ± 0.252	88.7242
		Increase or decrease	30.31%^**^	-54.16%^**^	7.26%^*^	51.63%^**^	14.50%^**^	-1.04%	-28.25%^**^	-	7.69%^*^
	*OPR4*	OPR4i	7.3477 ± 0.29	3.0894 ± 0.253	10.4371	19.9233 ± 5.850	26.4398 ± 3.586	16.0365 ± 2.200	18.3035 ± 2.500	1.5821 ± 0.399	82.2852
		Increase or decrease	-6.41%^*^	4.83%^*^	-3.34%	31.55%^**^	-6.26%^*^	-16.11%^**^	-8.14%^*^	-	-0.13%
		OPR4-OE	9.5807 ± 0.799	1.0081 ± 0.340	10.5888	20.9489 ± 3.030	31.4643 ± 0.984	19.8484 ± 7.047	15.3593 ± 1.484	0.2263 ± 0.282	87.8472
		Increase or decrease	22.03%^**^	-65.79%^**^	-1.94%	38.32%^**^	11.56%^**^	3.84%^*^	-22.91%^**^	-	6.62%^*^
T_2_	Contrast T_2_		9.09 ± 0.564	3.48 ± .0154	12.57	11.742 ± 2.154	27.486 ± 2.345	20.848 ± 1.253	18.165 ± 2.078	1.635 ± 0.178	79.876
	*OPR1*	OPR1i	8.463 ± 0.246	3.59 ± 0.784	12.053	9.23 ± 1.546	27.3638 ± 1.648	23.574 ± 5.791	18.205 ± 0.634	1.88 ± 0.481	80.2528
		Increase or decrease	-6.90%^*^	3.16%^*^	-4.11%^*^	-21.39%^**^	-0.44%	13.08%^**^	0.22%	14.98%^**^	0.47%
		OPR1-OE	10.217 ± 0.794	4.558 ± 0.254	14.775	9.897 ± 2.364	30.153 ± 0.994	21.122 ± 2.164	17.349 ± 1.424	1.645 ± 0.251	80.166
		Increase or decrease	12.40%^*^	30.98%^**^	17.54%^**^	-15.71%^**^	9.70%^*^	1.31%	-4.49%^*^	0.61%	0.36%
	*OPR2*	OPR2i	8.42 ± 0.284	4.048 ± 0.645	12.468	8.578 ± 3.045	27.71 ± 1.564	22.432 ± 1.578	17.839 ± 0.978	2.264 ± 4.165	78.823
		Increase or decrease	-7.37%^*^	16.32%^**^	-0.81%	-26.95%^**^	0.81%	7.60%^*^	-1.79%	38.47%^**^	-1.32%
		OPR2-OE	10.039 ± 0.451	5.191 ± 0.214	15.23	9.44 ± 1.231	30.056 ± 0.597	21.467 ± 0.146	15.291 ± 0.548	1.91 ± 0.841	78.164
		Increase or decrease	10.44%^*^	49.17%^**^	21.16%^**^	-19.60%^**^	9.35%^*^	2.97%	-15.82%^**^	16.82%^**^	-2.14%
	*OPR3*	OPR3i	8.856 ± 0.854	3.413 ± 0.847	12.269	8.259 ± 1.254	27.173 ± 0.914	24.266 ± 0.147	17.029 ± 0.934	2.244 ± 0.771	78.971
		Increase or decrease	-2.57%	-1.93%	-2.39%	-29.66%^**^	-1.14%	16.39%^**^	-6.25%^*^	37.25%^**^	-1.13%
		OPR3-OE	9.433 ± 1.349	3.884 ± 0.915	13.317 ± 1.015	10.063 ± 0.187	28.715 ± 0.942	20.721 ± 0.975	16.84 ± 0.943	2.147 ± 0.143	78.486
		Increase or decrease	3.77%	11.61%^**^	5.94%^*^	-14.30%^**^	4.47%^*^	-0.61%	-7.29%^*^	31.31%^**^	-1.74%
	*OPR4*	OPR4i	9.133 ± 0.643	3.454 ± 0.916	12.587 ± 0.241	7.517 ± 1.214	27.208 ± 0.914	24.708 ± 0.511	17.791 ± 0.261	2.012 ± 0.646	79.236
		Increase or decrease	0.47%	-0.75%	0.14%	-35.98%^**^	-1.01%	18.51%^**^	-2.06%	23.06%^**^	-0.80%
		OPR4-OE	8.757 ± 0.814	3.733 ± 0.646	12.49	11.382 ± 1.134	28.921 ± 0.615	20.511 ± 0.647	16.674 ± 1.057	1.611 ± 1.341	79.099
		Increase or decrease	-3.66%	7.27%^*^	-0.64%	-3.07%	5.22%^*^	-1.62%	-8.21%^*^	-1.47%	-0.97%

Note:*. *P* < 0.05; **. 0.01 < P < 0.05. The same as Table 6

The LA content in OPR3i decreased significantly; OPR3-OE significantly increased LA content; OPR4i significantly decreased LA content; and OPR4-OE increased LA content significantly.

Each copy of OPR-OE increased LA content, with an average increase of 12.56% in T_1_ generation and 7.185% in T_2_ generation. Subsequently, LA content in OPRi gene was significantly decreased, with an average decrease of 5.98% in T_1_ generation and 0.86% in T_2_ generation.

As shown in Table 5, oleic, linolenic, arachidonic, and erucic acids with the same variation trend as that of the fatty acid composition were selected for variance analysis. The results (Table 6) showed that the linolenic acid content in OPR1i significantly increased, while OPR2i significantly increased the linolenic acid content. Both OPR3-OE and OPR4-OE affected the content of arachidonic acid, which decreased significantly. In addition, OPR4i had no significant effect on the arachidonic acid content.

**Table 6:** Analysis of variance for the same trend of 35 s and RNAiTable

Strain		Oleic acid	Linolenic acid	Arachidonic acid	Erucic acid
T_1_	OPR1i	22.5651 ± 2.401	-	18.7597 ± 1.975	-
	OPR1-OE	20.5827 ± 1.609	-	18.3739 ± 3.331	-
	Mean square	19.65	-	0.744	-
	Mean square within group	4.177	-	7.496	-
	F value	4.705^*^	-	0.099	-
	OPR2i	16.8825 ± 1.533	17.4696 ± 1.08	-	-
	OPR2-OE	21.4321 ± 3.32	17.0149 ± 1.747	-	-
	Mean square	103.494	1.034	-	-
	Mean square within group	6.685	2.109	-	-
	F value	15.481^**^	0.49	-	-
	OPR3i	18.318 ± 2.432	17.5874 ± 1.266	19.3568 ± 0.831	-
	OPR3-OE	22.9638 ± 1.382	18.9167 ± 2.314	14.2964 ± 1.938	-
	Mean square	5.259	8.835	128.038	-
	Mean square within group	21.703	14.921	4.662	-
	F value	0.242	0.592	27.466^**^	-
	OPR4i	19.9233 ± 5.580	-	18.3035 ± 2.500	-
	OPR4-OE	20.9489 ± 3.030	-	15.3593 ± 1.484	-
	Mean square	107.917	-	43.342	-
	Mean square within group	46.968	-	4.225	-
	F value	2.298^*^	-	10.257^**^	-
T_2_	OPR1i	9.23 ± 1.546	23.574 ± 5.791	-	-
	OPR1-OE	9.897 ± 2.364	21.122 ± 2.164	-	-
	Mean square	1.439	4.824	-	-
	Mean square within group	1.0266	1.993	-	-
	F value	1.401	2.420^*^	-	-
	OPR2i	8.578 ± 3.045	22.432 ± 1.578	17.839 ±	2.264 ± 4.165
	OPR2-OE	9.44 ± 1.231	21.467 ± 0.146	15.291 ±	1.91 ± 0.841
	Mean square	0.00002	11.601	1.559	0.047
	Mean square within group	1.186	1.099	2.78	0.044
	F value	0.00002	10.560^**^	0.561	1.048
	OPR3i	8.259 ± 1.254	-	17.029 ± 0.934	2.244 ± 0.771
	OPR3-OE	10.063 ± 0.187	-	16.84 ± 0.943	2.147 ± 0.143
	Mean square	3.855	-	36.954	0.666
	Mean square within group	1.931	-	2.328	0.035
	F value	1.997	-	15.877^**^	18.799^**^
	OPR4i	7.517 ± 1.214	-	17.791 ± 0.261	-
	OPR4-OE	11.382 ± 1.134	-	16.674 ± 1.057	-
	Mean square	3.968	-	1.769	-
	Mean square within group	1.654	-	8.573	-
	F value	2.399^*^	-	0.206	-

We correlation analysis of FA in transgenic *Arabidopsis* seeds, according to the Table 7 shows that: In T_1_, the four *OPR* copies showed significant negative correlation between the OA, LA and linolenic acid content, OA mass fraction is higher, LA and linolenic acid mass fraction, the lower the relative; There was a positive correlation between LA and linolenic acid. Overall variation trend of LA and linolenic acid was basically the same. The mass fraction of linolenic acid was positively correlated with that of arachidonic acid. The stearic acid massed fraction and LA mass fraction had the same trend, and a larger trend with that of OA and linolenic acid. The mass fraction of palmitic acid was positively correlated with stearic acid, negatively correlated with OA (except *OPR1*), and positively correlated with LA and linolenic acid. The variation trend of FA in T_1_ and T_2_ generations is almost the same, there are also differences, and here was a significant negative correlation between LA and linolenic acid.

**Table 7:** FA correlation analysis of T1 and T2 seeds

Strain		Oleic acid / Linoleic acid	Oleic acid / Linolenic acid	Linoleic acid / Linolenic acid	Linolenic acid / Arachidonic acid	Palmitic acid / Stearic acid	Palmitic acid / Oleic acid	Palmitic acid / Linoleic acid	Palmitic acid / Linolenic acid	Stearic acid / Oleic acid	Stearic acid / Linoleic acid	Stearic acid / Linolenic acid
	Contrast	-0.373**	-0.489**	-0.626**	0.088	0.963**	-0.754**	0.891**	-0.203*	-0.549**	0.980**	-0.460**
T_1_	OPR1i	-0.788**	-0.837**	0.719**	0.866**	0.225*	0.878**	-0.699**	-0.570**	-0.448**	0.676**	-0.704**
	OPR1-OE	-0.142	-0.331*	0.501**	0.110	0.390*	0.515**	0.728**	0.683**	0.303**	0.360**	-0.503**
	OPR2i	-0.371*	-0.802**	-0.081	0.819**	0.526**	-0.974**	0.502**	0.894**	0.829**	0.050	-0.521**
	OPR2-OE	-0.852**	-0.916**	0.856**	-0.219**	0.778**	-0.427**	0.593**	0.611**	0.047	0.189	-0.188
	OPR3i	-0.541**	-0.743**	0.561**	0.504**	0.248**	-0.649**	-0.081**	0.461**	-0.515**	-0.064	0.039
	OPR3-OE	-0.550**	-0.579**	0.431**	0.184**	0.866**	-0.437**	0.888**	0.218	0.592**	0.735**	-0.089
	OPR4i	-0.967**	-0.970**	0.884**	0.901**	0.746**	-0.613**	0.596**	0.624**	0.265*	0.670**	-0.257*
	OPR4-OE	-0.772**	-0.505**	0.600**	0.053**	0.208*	-0.092**	-0.330*	-0.488**	0.216*	-0.263*	-0.510**
T_2_	OPR1i	-0.830**	-0.728**	-0.179*	0.234*	0.690**	-0.188	0.481**	-0.235*	-0.893**	0.328*	-0.642**
	OPR1-OE	-0.692**	-0.488**	-0.292*	0.120	0.811**	-0.669**	0.825**	-0.147	-0.810**	0.478**	-0.180
	OPR2i	-0.216	-0.681**	-0.556**	0.484**	0.938**	-0.322*	0.344*	-0.728**	-0.956**	0.260*	-0.838**
	OPR2-OE	-0.375*	-0.654**	-0.125	0.296*	0.143	-0.420**	0.971**	-0.270*	-0.328*	0.746**	-0.270*
	OPR3i	-0.416**	-0.271*	-0.405**	0.704**	0.885**	-0.053	0.726**	-0.722**	-0.938**	0.074	-0.859**
	OPR3-OE	-0.750**	-0.660**	-0.633**	0.149	0.350**	-0.744**	0.305*	-0.007	-0.202	0.283*	-0.341*
	OPR4i	-0.122	-0.689**	-0.197*	0.132	0.330	-0.713**	0.682**	-0.308**	-0.378**	0.166	-0.330*
	OPR4-OE	-0.785**	-0.611**	-0.551**	0.069	0.662**	-0.324**	0.790**	-0.180	-0.898**	0.771**	-0.180

Note: Lower triangle is r,upper triangle is p;“**” highly significant correlation(P < 0.01),“*” significant correlation(P < 0.05)

## Discussion

### miRNA expression and enrichment analysis

In recent years, the number of known miRNAs has increased continually, such as *A. thaliana* [43] and *Oryza sativa* [44, 45]. *Brassica napus L.* has a relatively high genome size and complexity [39], and the number and function of miRNAs in *Brassica napus L.* have not been adequately studied; which suggests that many miRNAs have not yet been discovered, especially in seeds.

In this study, miRNA libraries were constructed from self-pollinated seeds that were collected 20–35 d after pollination of rapeseed with high OA content in the near-isogenic lines. Clean reads exhibited 87.45% (A) and 88.26% (B) homology with the referenced genome sequences (Table 1). The small RNAs (24 nt) were most abundant in all the samples (Fig. 1A). The clustering analysis results showed that three samples of high or low OA contents were found in a cluster, revealing that these miRNAs might have similar biological functions (Fig. 1B). A total of 21 differentially expressed miRNAs were detected (Table 2, Supplemental Table 2), including 9 (42.86%) up-regulated and 12 (57.14%) down-regulated genes (Fig. 1C) using GO and KEGG pathways. In our study, *bna-miR156b* > *c* > *g* may be involved in fatty acid metabolism. Genes related to Cd stress have previously been discovered using GO and KEGG pathways, such as *BNPCS1*, *BNGSTU12,* and *BNGSTU5* [46]. Jian et al. (2018) found a total of 13 differentially expressed miRNAs were confirmed by RT-qPCR, and a hypothetical model of cadmium response mechanism in *B.napus* was proposed on this basis [47].

### Multi copy phenomenon of *OPR* genes

We found that *OPR* gene has four copies in rapeseed, with *OPR1* and *OPR4* located in the C genome and *OPR2* and *OPR3* located in the A genome. The *OPR* gene has been identified in several species and there are often multiple copies. Three *OPRs* were found in A. thaliana and tomato [48], 6 in peas [49], and 8 in corn [50]. Meanwhile, rice comprised 13 *OPRs* [51] and wheat had 48 *OPRs* [52]. Multi-copy genes are ubiquitous in plants and play an important role in maintaining plant genetic stability; However, they have hindered molecular breeding research. The loss of a few copies of gene function often does not cause phenotypic changes, and the probability of simultaneous mutation of multiple copy sites is too low to create a gene family or multiple copies of genes change simultaneously [53]. Rapeseed is an allotetraploid with many multi-copy genes. Conventional molecular breeding research methods are difficult to obtain phenotypic multi-copy gene mutants. Handa (2003) found that the main DNA sequence of the protein coding region was highly conserved between rapeseed and *A. thaliana* [54]. Transformation with *A. thaliana* as a receptor is helpful to study the function of multi-copy genes in rapeseed. In this study, we found four copies of *OPR* gene in rapeseed. Based on these multi-copy genes, we transformed four copies of *OPR* genes in *A. thaliana*.

### Regulation function of *OPR* genes to fatty acid

**Fig. 6:**
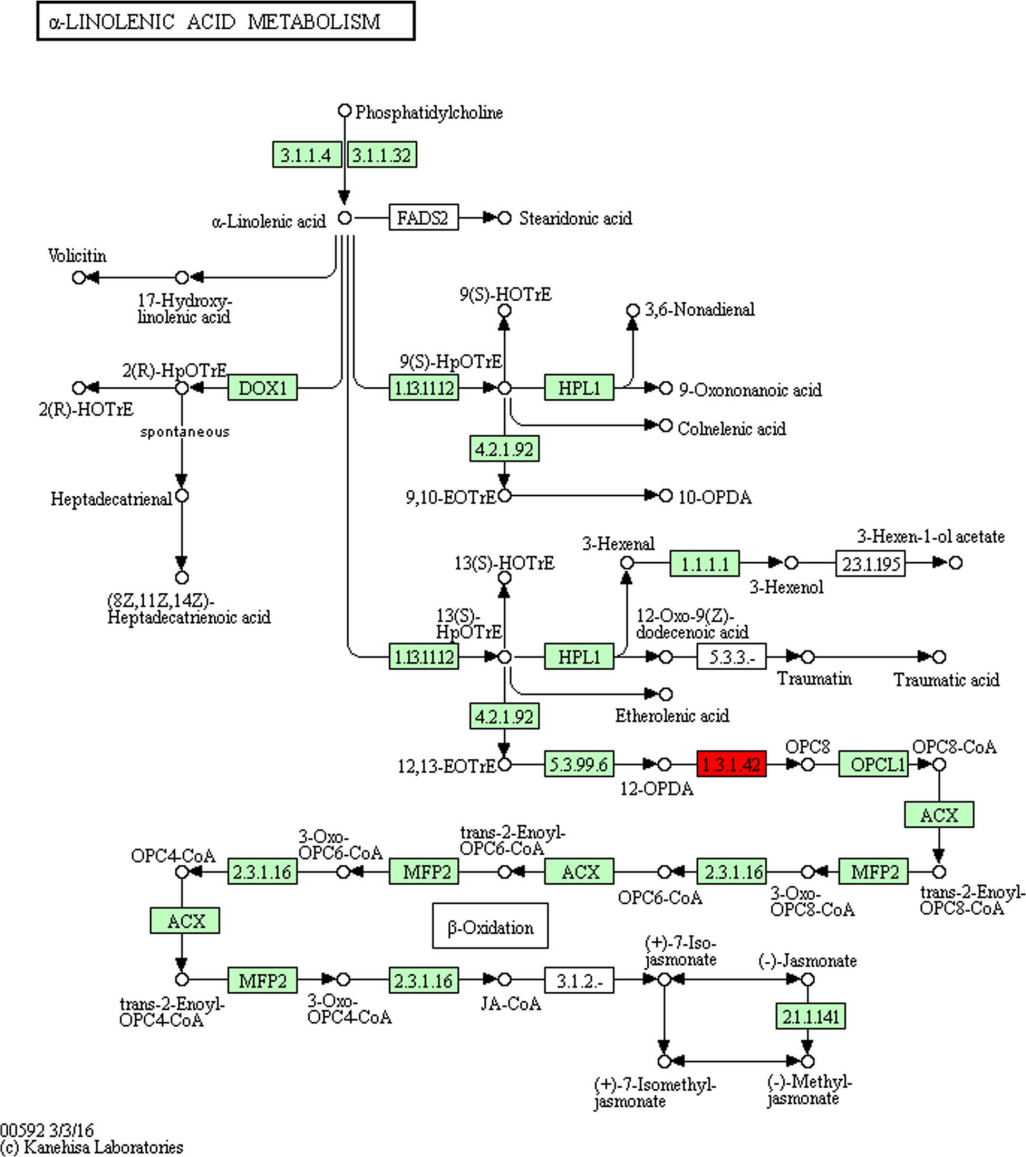
*Bna00592* KEGG patway network

In this study, we found that *OPR* genes may affect the metabolism of LA and each copy was transferred separately into *A. thaliana*. The LA content of OPR-OE transgenic plants was significantly increased (T_1_ 12.56%, T_2_ 7.185%), while the LA content of OPRi transgenic plants was significantly decreased (T_1_ 5.98%, T_2_ 0.86%). These results have rarely been reported before. However, it has been described that the *ClOPR* genes, particularly *ClOPR2* and *ClOPR4*, significantly upregulated by exogenous jasmonic acid, salicylic acid, and ethylene treatments in watermelon [55]. Virus-induced gene silencing (VIGS) analysis suggested that knockdown of *GhOPR9* could increase the susceptibility of *cotton* to V. dahliae infection [56]. *OPR* gene was cloned from *Oryza sativa*; the overexpression of *OPR* genes was found to enhance the stress resistance of tobacco to heavy metal Cd^2+^ [57]. Previous studies concluded that *OPR* genes were widely involved in abiotic stress processes [58]. In addition, *OPR* genes involved in fatty acid β oxidation, cilinolenic acid reduction, and the octadecanoic acid metabolic pathway [59]. However, there were few reports on *OPR* genes regulating OA, LA, or saturated fatty acid synthesis. In our study, we found that *OPR* genes directly affect the synthesis of LA and indirectly affect the content of other FA (Fig. 6**)**, which is consistent with the theoretical pathway wherein *OPR* genes regulate jasmonic acid synthesis using alpha-linolenic acid (18: 3) [59, 60].

According to the Table 7, The four *OPR* copies showed significant negative correlation between the OA, LA and linolenic acid content, which was consistent with Zhao J (2008) [61] and Yan (2012) [62]; The stearic acid massed fraction and LA mass fraction had the same trend, and a larger trend with that of OA and linolenic acid. This is consistent with findings of Shang et al. [63]. Linolenic acid content is not only affected by OA and LA, but also affected by another independent gene system, which was consistent with Kondra (1975) [64]. The variation trend of FA in T_1_ and T_2_ generations is almost the same, there are also differences, and here was a significant negative correlation between LA and linolenic acid. In the meantime, there are many researchers believed that FA content of rapeseed was controlled by maternal genotype, and there were interaction, additive and non-additive gene effects between genotype and environment [65–67].

*A. thaliana* and rapeseed are both cruciferous plants and current studies have shown that their gene functions are basically the same, it is of practical significance to study in model plants [54]. We found the optimizing quality FA in *A. thaliana* can be changed by regulating *OPR* genes. *OPR* may be involved in regulating LA synthesis and improving fatty acid composition in rapeseed. This is the first study which discovered that the *OPR* gene can regulate LA metabolism. Therefore, this study is a good reference for studies researching the molecular mechanism of LA synthesis and molecular breeding in rapeseed.

In this study, 20 pathways were enriched using the KEGG pathway through high-throughput sequencing, of which 15 may be involved in the regulation of fatty acid metabolism. The reliability of the results was verified by performing the RT-qPCR analysis, which provided a basis for subsequent functional verification. We excavated a target gene *OPR*, *bna-miR156b* > *c* > *g*, from rapeseed that may be related to fatty acid synthesis and identified the function of *OPR* genes through transformation of *A. thaliana.* The LA content of OPR-OE transgenic plants significantly increased (T_1_ 12.56%, T_2_ 7.185%), and the LA content of OPRi transgenic plants significantly decreased (T_1_ 5.98%, T_2_ 0.86%). In addition, by performing a bioinformatics analysis, we found four copies of the *OPR* gene in the cytoplasm that were located on chromosomes A and C. In this study, by detecting the fatty acid content of different generations of transgenic *A. thaliana*. OA and LA, linolenic acid content showed significant relationship, LA can be affected by FAD-related gene regulation and the environment. We found the four copies of *OPR* gene that can directly affect LA content and indirectly affect other high quality FA were discovered for the first time. These results can be used in breeding programs aimed at optimizing fatty acid profiles in rapeseed [68].

## Methods

### Plant materials and growth conditions

The near-isogenic rapeseed lines with high (81.4%) and low (56.2%) OA contents were provided by Rapeseed Molecular Breeding of Hunan Agricultural University, high OA rapeseed materials became known as HOAR and low OA rapeseed materials became known as LOAR, the strain was originally cultivated by National Oil Improvement Center of Hunan Agricultural University. The materials exhibited stable traits and were planted in the experimental field of Hunan Agricultural University, China (Changsha, China) with standard agronomic methods [69]. The seeds 20 d after pollination were quickly frozen in liquid nitrogen and stored at -80 °C for subsequent studies [70]. The sample treatment method adopted in this study is the same with our previous study [71].

The seeds of wild-type (WT) *A. thaliana* (ecotype: Columbia) were bought from Think Gene Biological Technology Co., LTD, Shanghai, China. Plants were grown under greenhouse conditions: 24 °C with a photoperiod of 18.5 h-light/5.5 h-dark, with a light intensity of 6500 lx.

## Methods

### sRNA library construction and high throughput sequencing

Total RNA was extracted from frozen seeds using the Trizol reagent (Sigma Aldrich, St. Louis, MO, USA), according to the manufacturer’s instructions. The quality and quantity of the purified RNA were assessed using an Agilent 2100 Bioanalyzer (Agilent Technologies, Palo Alto, Santa Clara, USA) and RNA 6000 nanokit (Agilent Technologies, Palo Alto, Santa Clara, USA). sRNAs with lengths of 18–30 nt were separated and purified using 15% denaturing polyacrylamide gel electrophoresis. Consequently, sRNAs fractions were ligated to the 5′ lectrophoresters using T4 RNA ligase (Epicentre, America). The adapter-ligated fragments were then reverse transcribed and amplified by performing PCR with a pair of adapter complementary primers. These PCR products were purified and sequenced using IlluminaHiseq XTEN (Illumina, USA). Construction of the sRNA libraries and deep sequencing were carried out by Oebiotech Genomics (Shanghai, China).

### Bioinformatic analysis of the sRNAs sequencing data

Clean reads were generated after eliminating the low-quality reads, poly As, reads smaller than 18 nt, and gener adaptor contaminants and subsequently inserting nulls. Using bowtie software [72], the clean reads were aligned against the NCBI Gen Bank [73], *Brassica napus* oilseed genome [39], and Rfam databases (version 10.0). Reads annotated into the noncoding RNA categories, including rRNA, tRNA, snRNA, and snoRNA were filtered. The remaining sRNA sequences were aligned against the mi RBase21.0 [74]. The nearly matched sequences (less than two mismatches) were considered to be the known miRNAs. Target miRAN will computationally predict small RNA binding sites on target transcripts from a sequence database, this is done by aligning the input small RNA sequence against all transcripts, followed by site scoring using a position-weighted scoring matrix [75]. First, used ‘targetfinder’ software, set threshold -C <  = 4, predicting the miRNA target genes, we got 64 target genes with miRNA156; and then, to GO and KEGG analysis (*P*-value ≤ 0.05) of 64 target genes, we found there are six of them related to the metabolism of FA. The unannotated sRNAs were further analyzed to predict the novel miRNAs using the Mirdeep 2 software. The secondary structures of premiRNAs were also predicted using the RNAfold software.

To reveal the continuous changes in expression of miRNAs during the biosynthesis process, the variation in expression was analyzed in immature seed libraries of HOAR. The frequency of miRNAs was normalized as transcripts per million (TPM) for further analysis. In addition, miRNAs were assessed using the negative binomial distribution test, with *P*-value ≤ 0.05 and absolute value of log2 (treatment/control: LOAR/HOAR) > 1.5 being considered as differentially expressed. Moreover, the similarity between samples was investigated by the clustering method. The Blast 2 GO software with default parameters was applied to determine the functional annotation and categorization of the target genes [76]. The KEGG (Kyoto Encyclopedia of Genes and Genomes) and InterPro databases were also searched with an evalue of 1e^−10^ [77–79].

### Real time quantitative polymerase chain reaction (RT-qPCR) validation of differentially expressed miRNAs

To validate the expression of differentially expressed miRNAs, all annotated miRNAs were selected for RT-qPCR validation using the poly (T) adaptor RT-qPCR method [80]. The RT-qPCR amplifications were performed according to an established procedure [81]. 5S rRNA was used as an internal reference gene of miRNA and two primers were used: F: 5'-CTCGGCAACGGATATCTCG-3' and R: 5'-CTAATGGCTTGGGGCG-3'. The internal reference gene of the miRNA target gene was *UBC9* and two primers were used: F: 5'-TCCATCCGACAGCCCTTACTCT-3' and R: 5'-ACACTTTGGTCCTAAAAGCCACC-3'. All reactions were performed in triplicate. The RT-qPCR was conducted on the Step One Plus RT-qPCR System (ABI, America). The relative expression was calculated using the 2 − ΔΔCT method [82]. Statistical analyses consisted of analysis of variance and Fisher’spost-hoc tests. A p-value < 0.05 was considered statistically significant. Details of the RT-qPCR primers were provided in Table 2.

### Cloning of *OPR* genes in rapeseed and bioinformatic analysis

Four copies of *OPR* genes were found using miRNA sequencing homologous cloning. We used material HOAR (high OA rapeseed materials materials, B) to clone, and the total RNA was extracted from the rapeseed leaf samples using the CTAB method adopted by Niu et al. (2018) and Meng et al. (2006) [83, 84], with minor modifications. cDNA was produced by implementing reverse transcription, which was followed by PCR amplification (Table 8). According to each copy sequence of *OPR*, the physical and chemical properties of corresponding proteins were analyzed by using online websites such as the ExPASY-Protparam Tool and modifying the methods described by Li et al. (2019) and Sun et al. (2016) [85, 86].

**Table 8:** Primers for OPR genes amplification

Target ID	Primer name	Primer sequence (5'-3')
*GSBRNA2T00012422001 (OPR1)*	*OPR1* BamHI-F	TCTGATCAAGAGACAGGATCCATGGAAAATGCAGTAGCGAAAG
	*OPR1* SalI-R	CATCGGTGCACTAGTGTCGACTTAAGCTGTTGATTCAAGAAAAG
*GSBRNA2T00135385001 (OPR2)*	*OPR2* BamHI-F	TCTGATCAAGAGACAGGATCCATGGAAAACGTAGTAACGAAAC
	*OPR2* SalI-R	CATCGGTGCACTAGTGTCGACTTAACTAGCTGTTGAATCAAG
*GSBRNA2T00082938001 (OPR3)*	*OPR3* BamHI-F	TCTGATCAAGAGACAGGATCCATGGAAAATGCAGTAGCGAAAC
	*OPR3* SalI-R	CATCGGTGCACTAGTGTCGACTTAAGCTTTTGATTCAAGAAAAG
*GSBRNA2T00094910001 (OPR4)*	*OPR4* BamHI-F	TCTGATCAAGAGACAGGATCCATGGAAAACGTAGTGACGAAAC
	*OPR4* SalI-R	CATCGGTGCACTAGTGTCGACTTAACTAGCTGTTGAATCAAG

Note: the underline indicates the restriction site. the same as Table 9

Bioinformatic analysis of *OPRs*: The ExPASy-ProtParam tool website (https://web.expasy.org/protparam/) predicted the primary structure of the protein. The subcellular localization of OPR proteins were performed using the PredictProtein SOPMA website (http://www.predictedprotein.org/). TMHMM Server, v. 2.0 (http://www.cbs.dtu.dk/services/TMHMM/) was usedto analyze the transmembrane helix region of proteins. The SOPMA website (https://npsa-prabi.ibcp.fr/cgi-bin/npsa_automation.pl?Page=npsa_sopma.html) predicted the secondary protein structure. The tertiary structure model of each copy of OPR protein was constructed using the SWISS-MODEL website (https://swissmodel.expasy.org/).

### Vector construction and transformation of *A. thaliana*

Specific RNAi primer (Table 9) cDNA was used as a template. The base before the 5'-end enzyme digestion site was used for the construction of recombinant plasmids (the same below), according to the method adopted by Li et al. (2021) with minor modifications [87]. The cloned *OPR* sequence was digested with *BamH* I and *Sal* I (Table 8) and recombinant with pCAMBIA1300-35 s vector (Kangyan Corporation, China) to construct pCAMBIA1300-35 s-*OPR* vector.

**Table 9:** RNAi primers

Gene name	Primer name	Primer sequence(5'-3')
*OPR1*	*OPR*1 Ri-PBF	GAGTCTCTCTGCAGGGATCCATGGAAAATGCAGTAGCG
	*OPR*1 Ri-SXR	TTTCCAGGTCGACTCTAGAACCTTTGGCATGAACAGC
*OPR2*	*OPR*2 Ri-PBF	GAGTCTCTCTGCAGGGATCCATGGAAAACGTAGTAACG
	*OPR*2 Ri-SXR	TTTCCAGGTCGACTCTAGAGCCTTTTGCATGAACAGC
*OPR3*	*OPR*3 Ri-PBF	GAGTCTCTCTGCAGGGATCCATGGAAAATGCAGTAGCG
	*OPR*3 Ri-SXR	TTTCCAGGTCGACTCTAGAACCTTTGGCATGAACAGC
*OPR4*	*OPR*4 Ri-PBF	GAGTCTCTCTGCAGGGATCCATGGAAAACGTAGTGACG
	*OPR*4 Ri-SXR	TTTCCAGGTCGACTCTAGAACCTTTGGCATGAACAGC

Similarly, the pCAMBIA1300-RNAi-*OPR* vector (Kangyan Corporation, China) was reconstructed using a double digestion process in vitro (Table 9) by following the methods of Yang et al. (2012) and Qu et al. (2017) with minor modifications [6, 29]. pCAMBIA1300-35 s-*OPR* and pCAMBIA1300-RNAi-*OPR* vectors were transformed into *A. thaliana* because of inflorescence infection to explore the function of each copy by using the inflorescence infection method of Clough and Bent (1998) [41], with minor modifications.

### Fatty acid detection and statistical methods

Using Agilent Technologies 7890B gas chromatograph, MSD detector, 5977A chromatographic workstation, and DB-23 capillary chromatographic column, sample size 1μ, Heating procedure: initial holding time 10 min, initial temperature 180 °C, 20 °C / min, rising to 250 °C, according to the fatty acid detection method of Mao et al. (2020) [42], with minor modifications.

Microsoft Excel 2010 was used to collate the data and one-way analysis of variance was used for analysis [88].

## Supplementary Information

**Additional file 1.**

## Data Availability

Data of RNA-seq in this study is available in NCBI with accession number PRJNA760803 that are publicly accessible at https://www.ncbi.nlm.nih.gov/sra/PRJNA760803.

## Abbreviations

OPR: 12-Oxo-phytodienoic acid reductase
LOAR (A): Low Oleic Acid Rapeseed (81.4%) materials
HOAR (B): High Oleic Acid Rapeseed (56.2%) materials
RT-qPCR: Quantitative Real-time PCR
OPR-OE: *OPR* Over-Expression strain
OPRi: *OPRi* RNA-interference strain
FA: Fatty acids
OA: Oleic acid
LA: Linoleic acid
ALA: α-Linolenic acid
GLA: γ-Linolenic acid
