# Supplementary material for: Clone and Function Verification of the OPR gene in Brassica napus Related to Linoleic Acid Synthesis
Source: BMC Plant Biol. 2022 Apr 12;22:192. doi: 10.1186/s12870-022-03549-1 (PMC9003975; doi:10.1186/s12870-022-03549-1)

**Figure 1** Sample size, sample-to-sample cluster and miRNAs cluster, GO enrichment analysis:

(A) Size distribution of the clean reads

(B) Results of sample-to-sample cluster analysis, Sample-A: Low oleic acid rapeseed materials; Sample-B: High oleic acid rapeseed materials;

(C) Cluster distribution diagram of the different miRNAs;


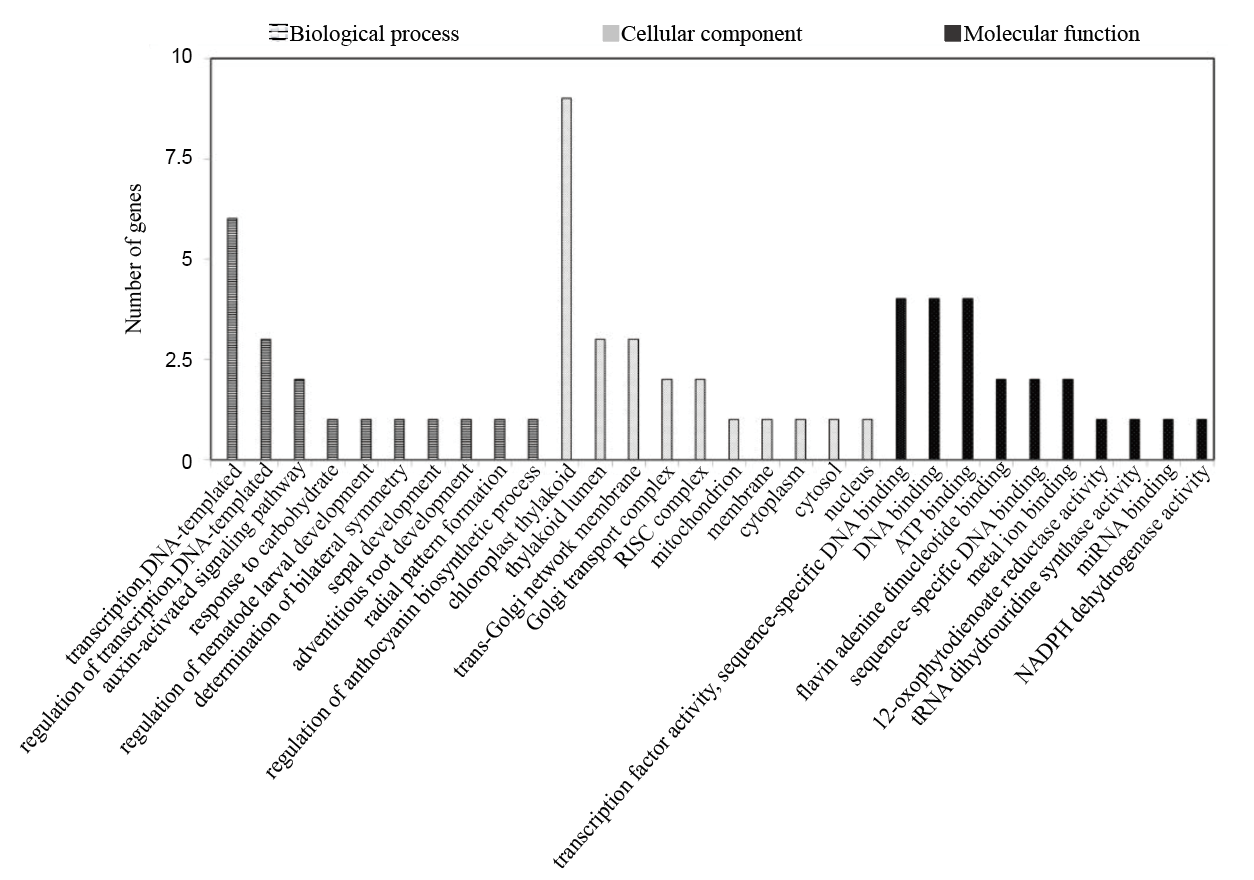
(D) GO enrichment analysis TOP10.

**Figure 2** KEGG enrichments, RT-PCR results of miRNAs and the expressions of miRNAs and their target genes:


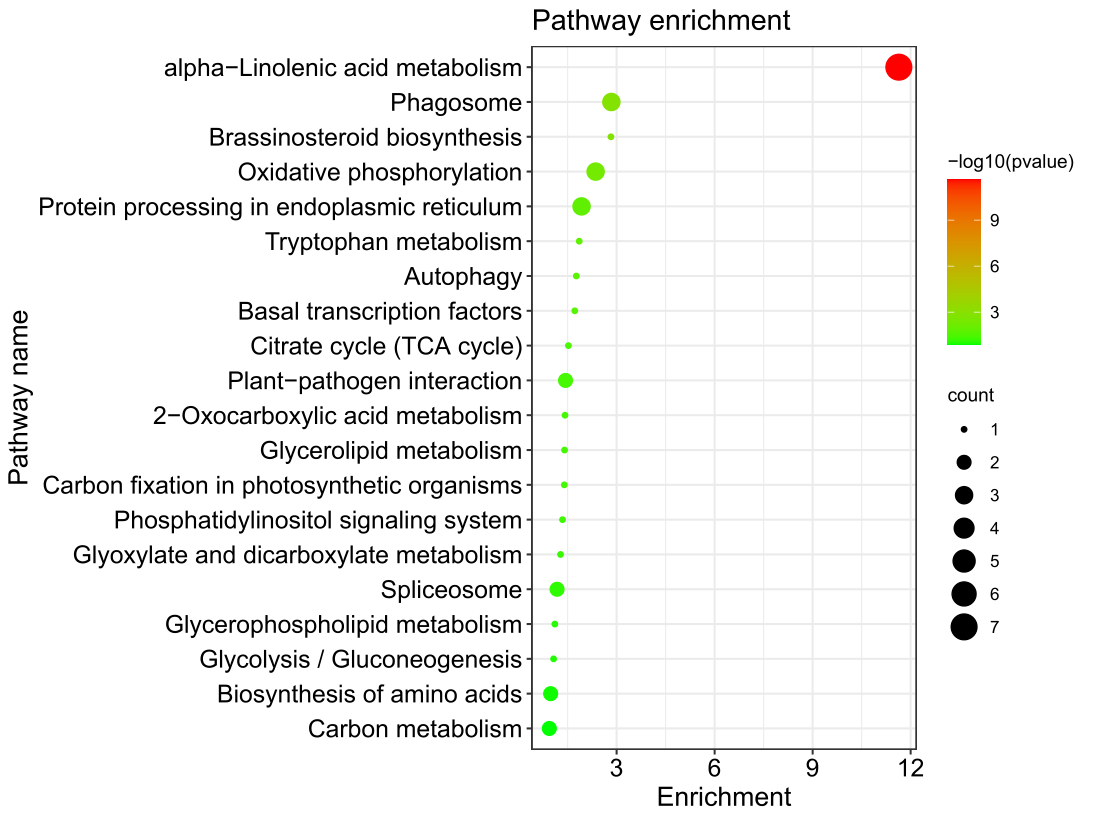


(A) Top 20 KEGG enrichments;


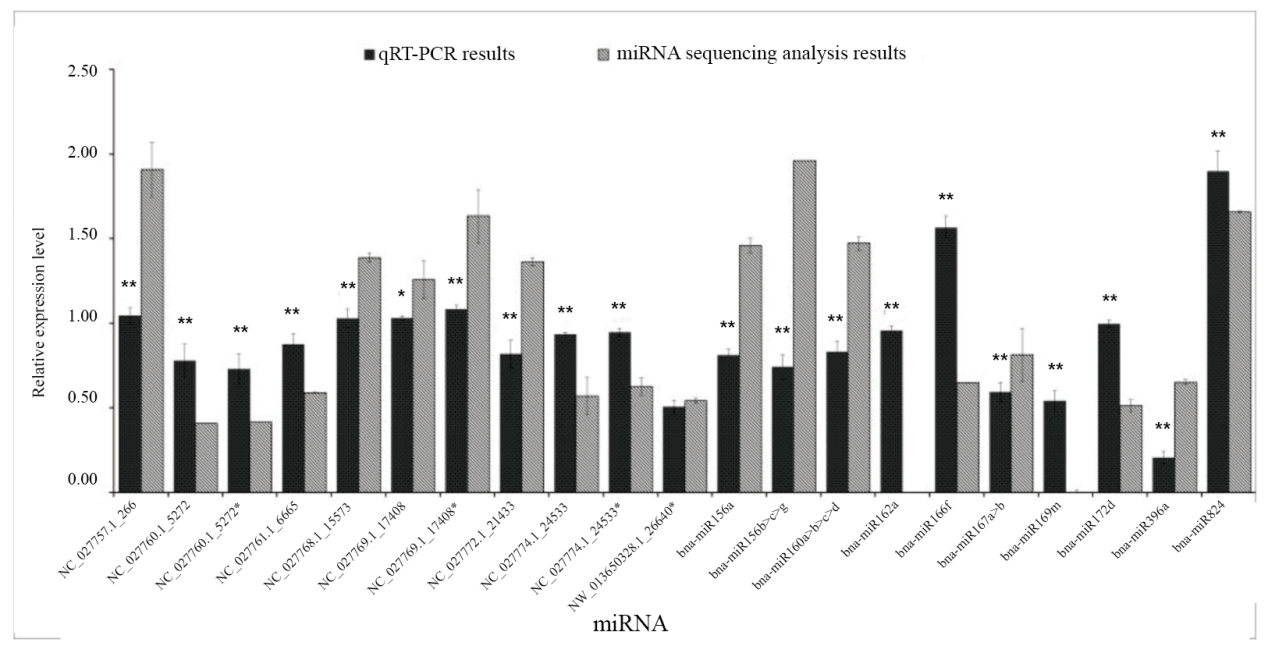


(B) The RT-PCR results of 21 differential miRNAs. The results of low oleic acid rapeseed materials were used as control. (* and ** signify the difference level of P < 0.05 and P < 0.01, respectively);


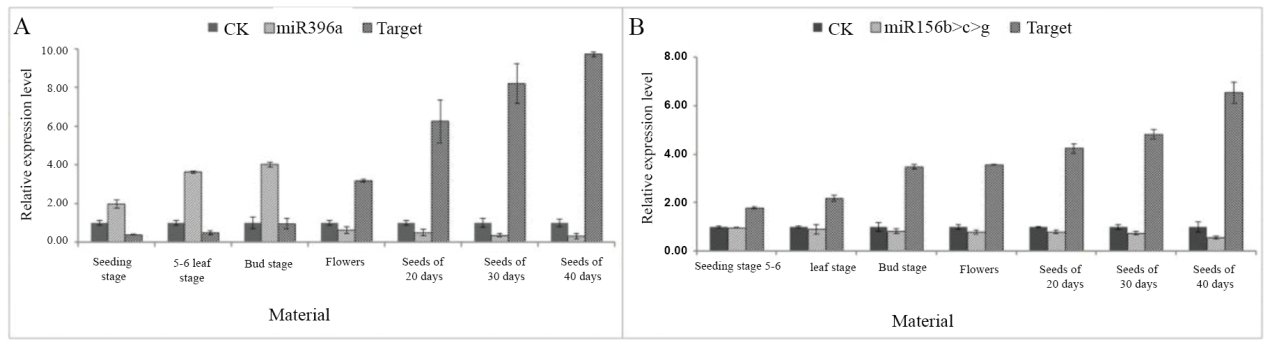


(C) The expressions of miRNAs (miR396a, miR1566b>c>g) and their target genes. The expression of miRNA and its target gene were used as controls.

**Figure 3** *OPR* genes PCR amplification, sequence and homology and the tertiary structure of protein analysis, RNAi fragment PCR amplification:


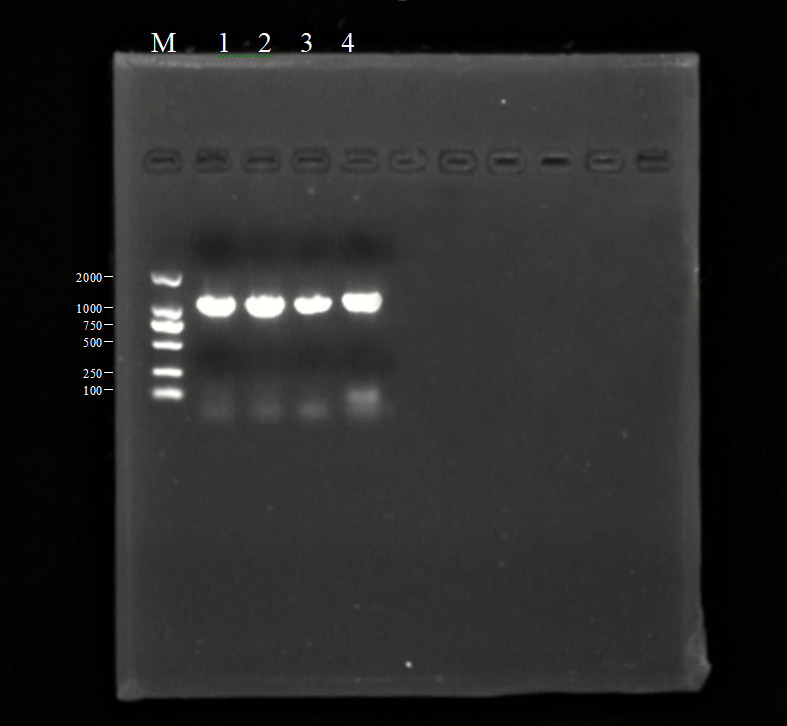


(A) Target gene PCR amplification (M: *Trans* 2000 bp; 1: *OPR1*; 2: *OPR2*; 3: *OPR3* 4: *OPR4*);


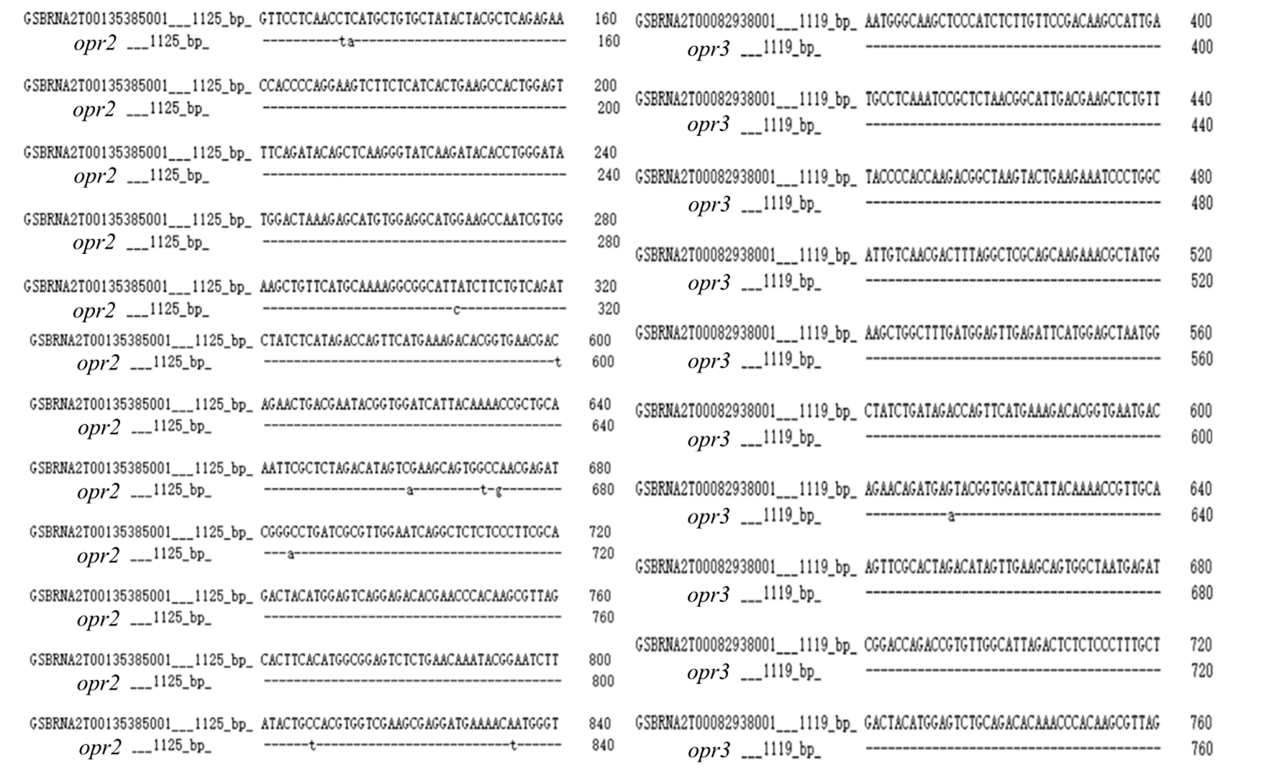


(B) Sequence alignment with the sequence published in Brassica Database;


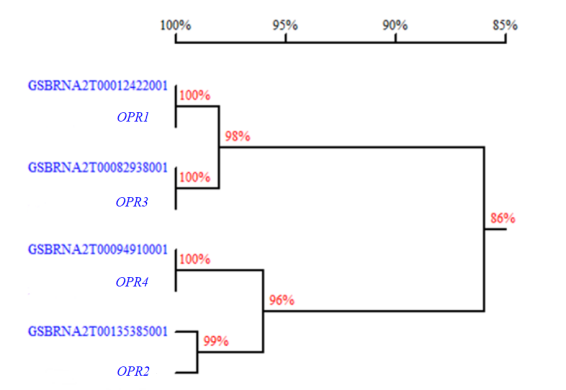


(C) Sequence homology analysis with Brassica Database;


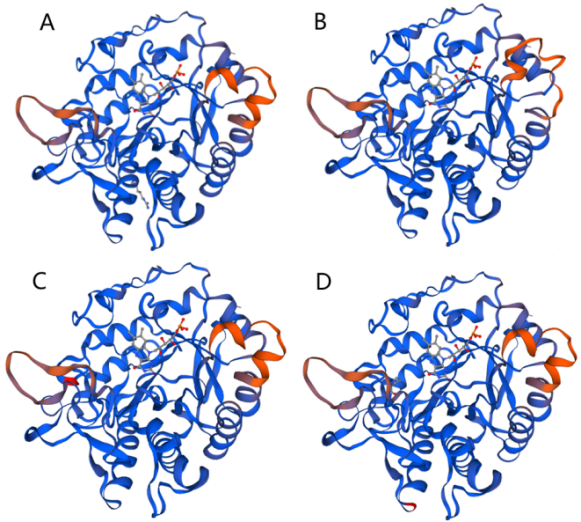


(D) Tertiary structure of protein (A. *OPR1*; B. *OPR2*; C. *OPR3*; D. *OPR4*);


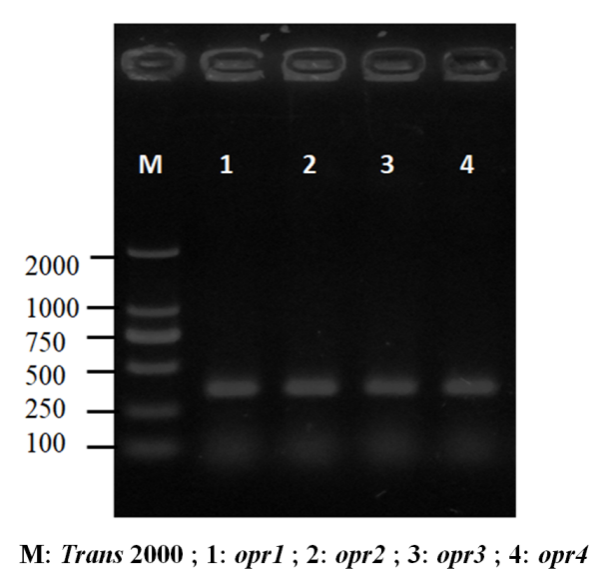


(E) Objective Gene RNAi fragment PCR electrophoresis map.

**Figure 4** *OPR* genes 35s and RNAi recombinant vector map:


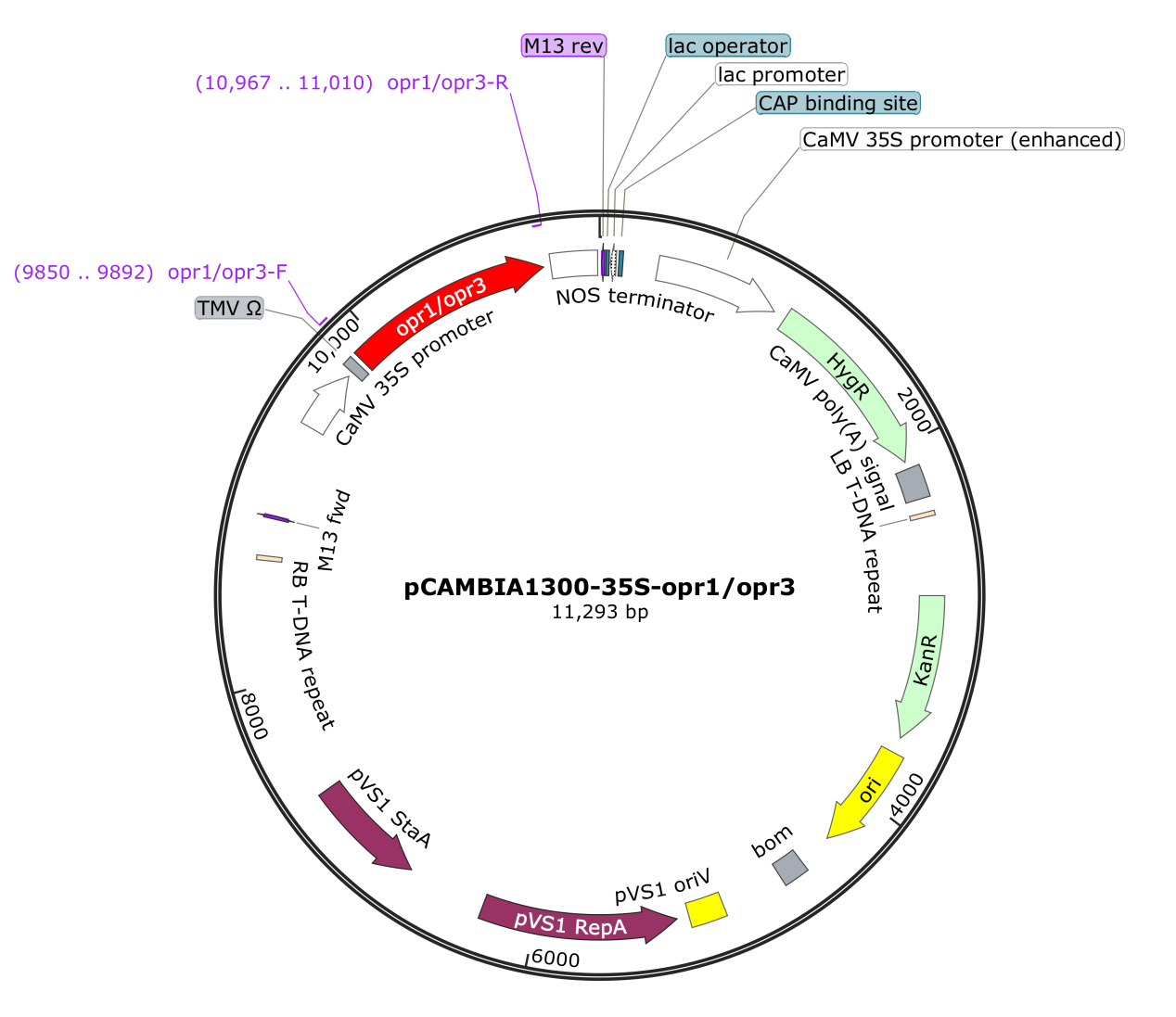


(A) pCAMBIA1300-35s-*OPR1*/*OPR3* recombinant vector;


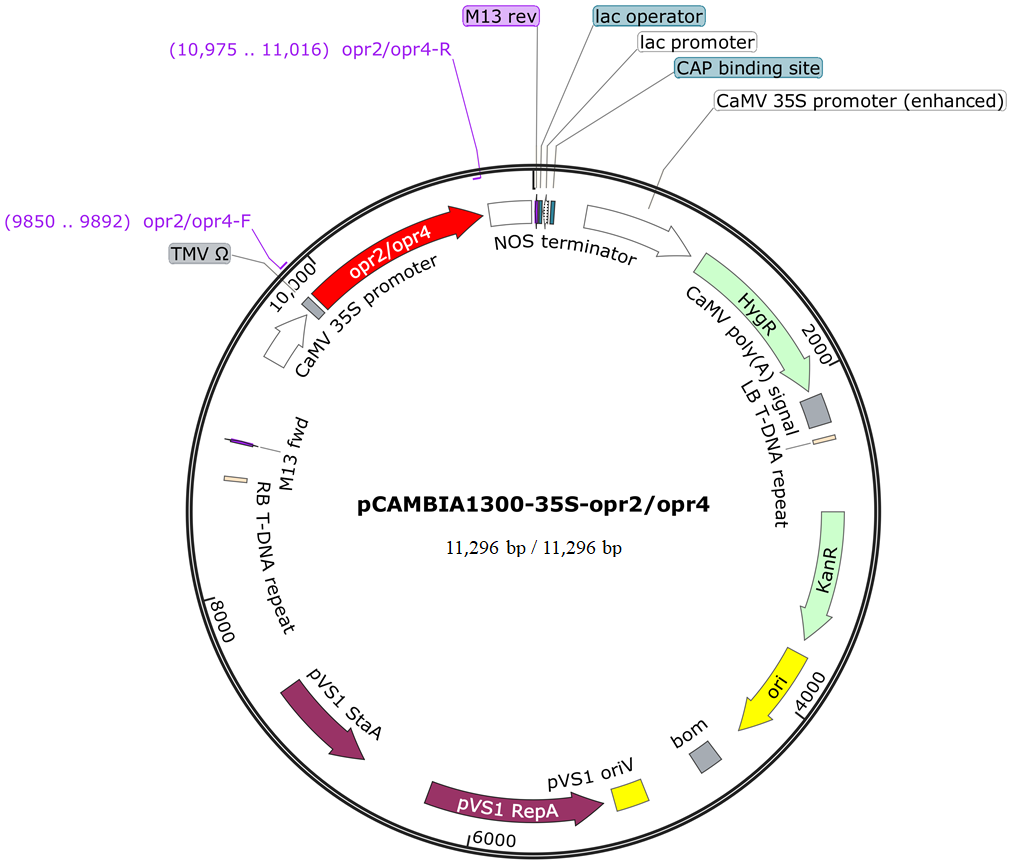


(B) pCAMBIA1300-35s-*OPR2*/*OPR4* recombinant vector;


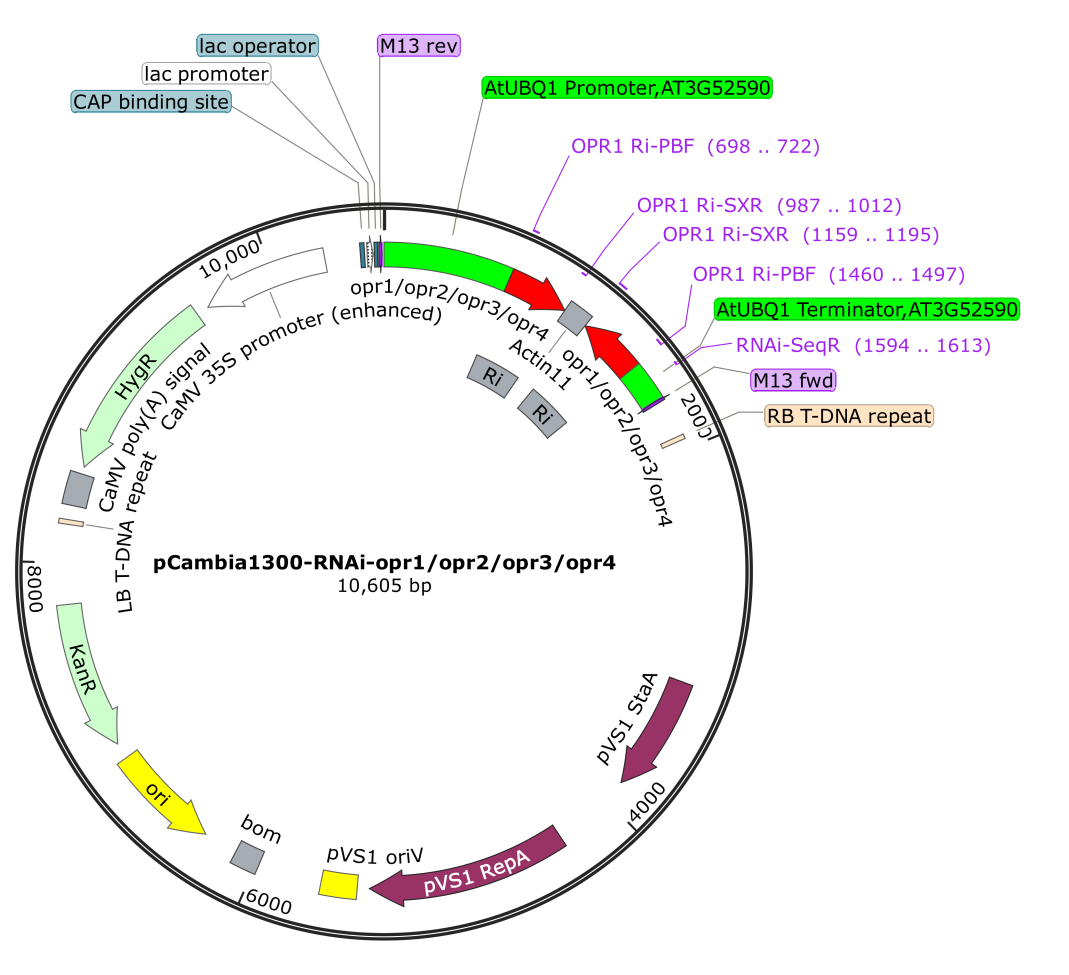


(C) pCAMBIA1300-RNAi-*OPR1*/*OPR2*/*OPR3*/*OPR4* recombinant vector.

**Figure 5** Screening and identification of transgenic *A. thaliana:*


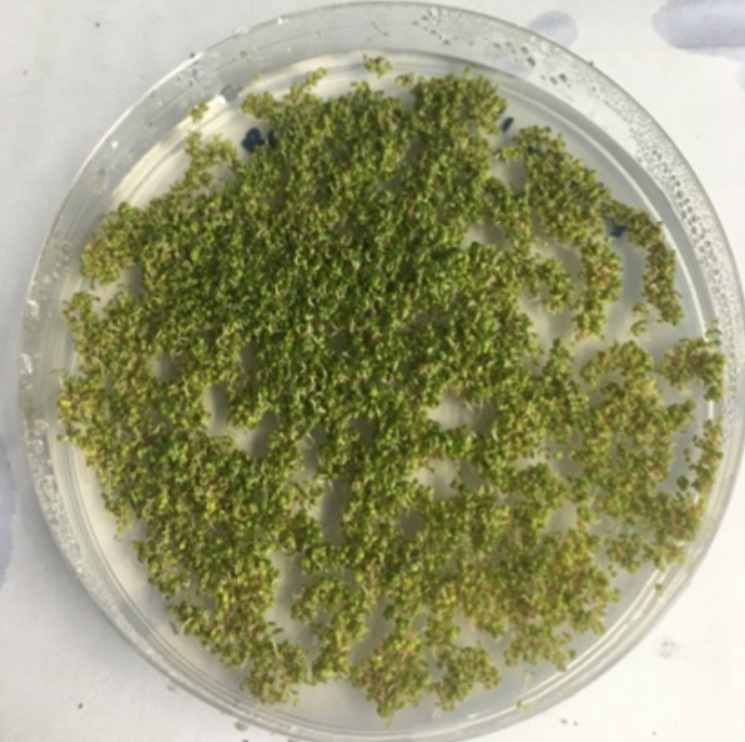


a: OPR1-OE

(A) Hygromycin screening of transgenic *A. thaliana*;


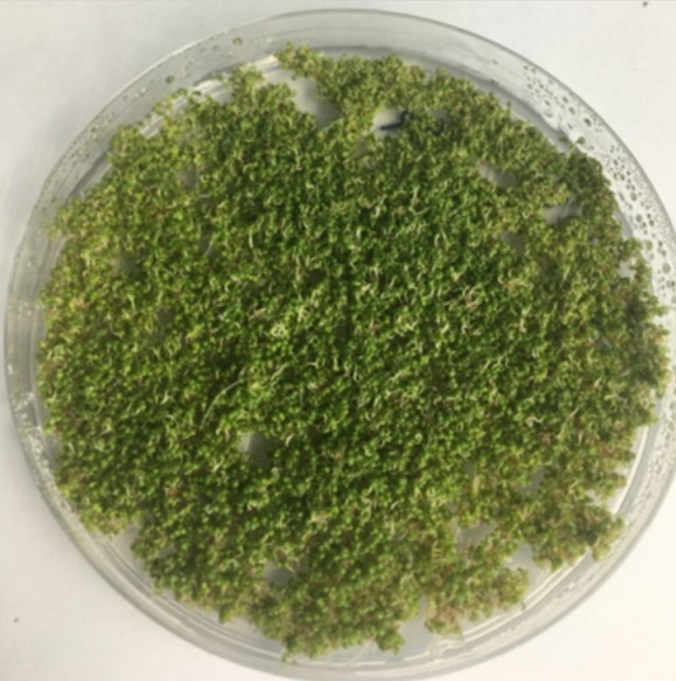


b: OPR1i

(A) Hygromycin screening of transgenic *A. thaliana*.


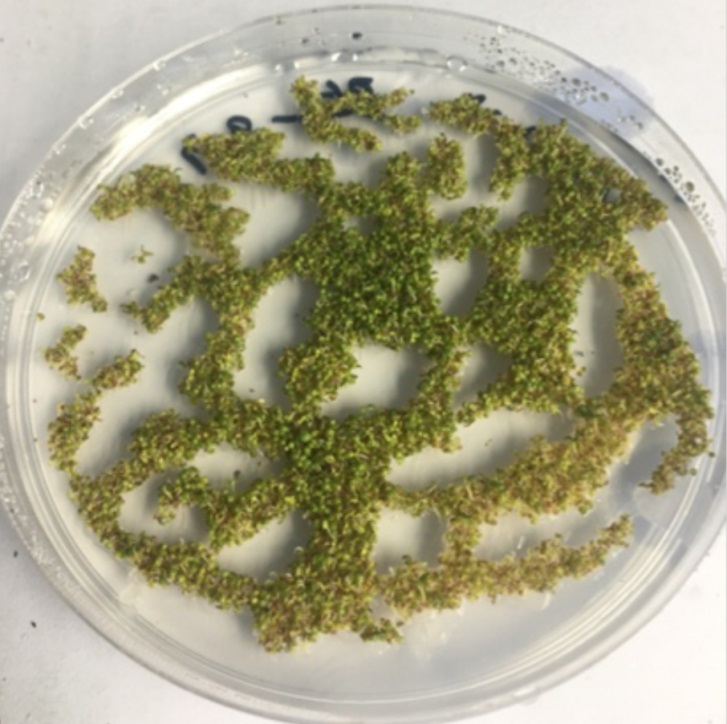


c: OPR2-OE

(A) Hygromycin screening of transgenic *A. thaliana*;


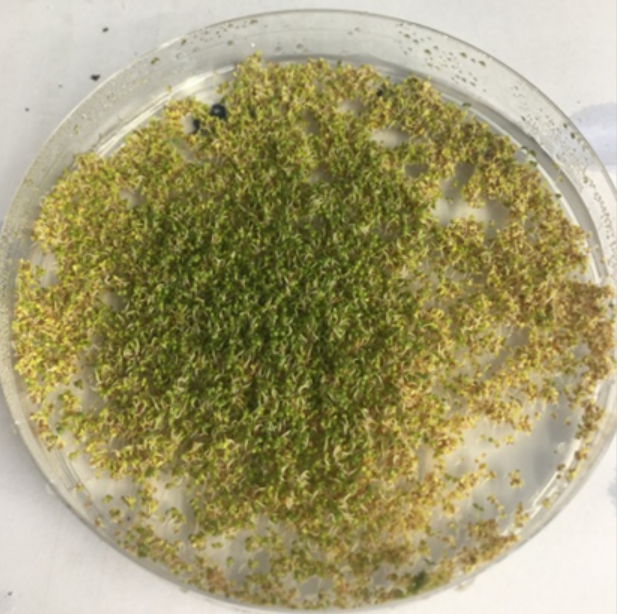


d: OPR2i

(A) Hygromycin screening of transgenic *A. thaliana*.


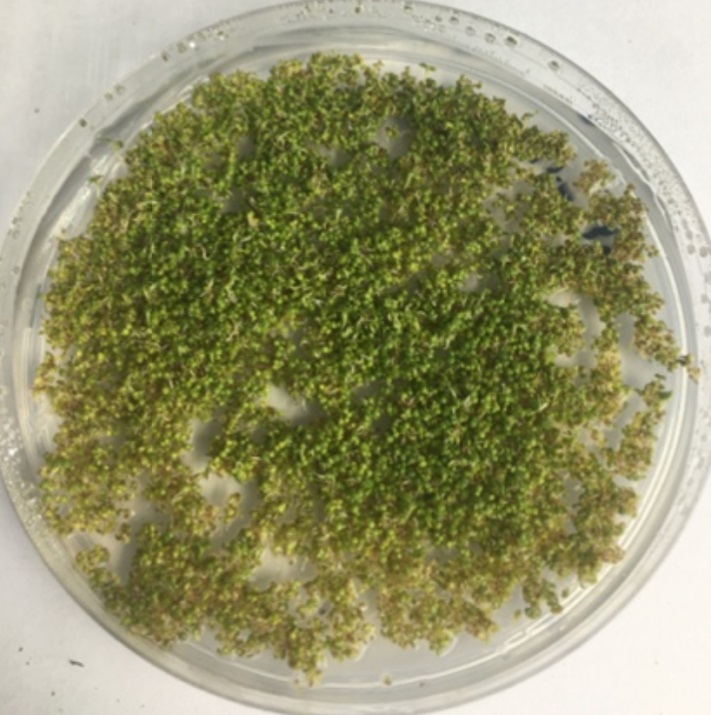


e: OPR3-OE

(A) Hygromycin screening of transgenic *A. thaliana*;


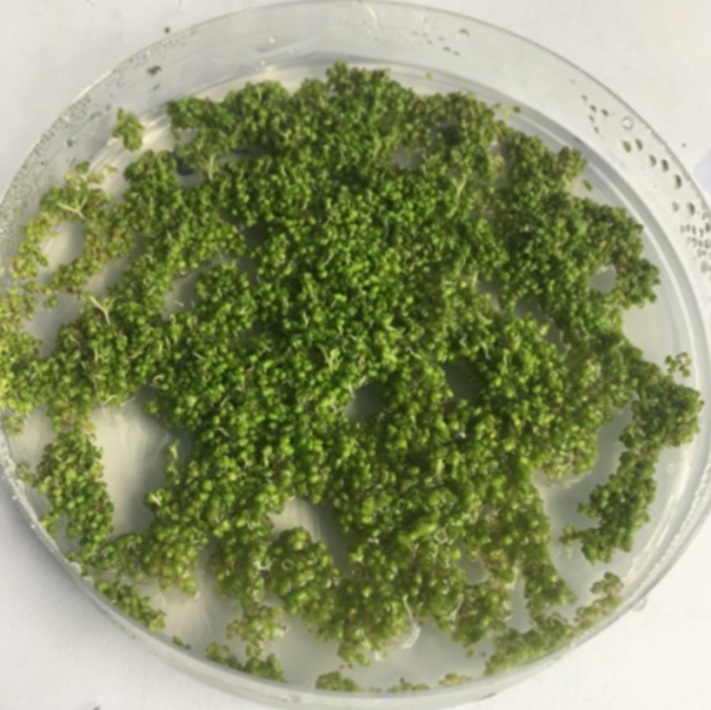


f: OPR3i

(A) Hygromycin screening of transgenic *A. thaliana*.


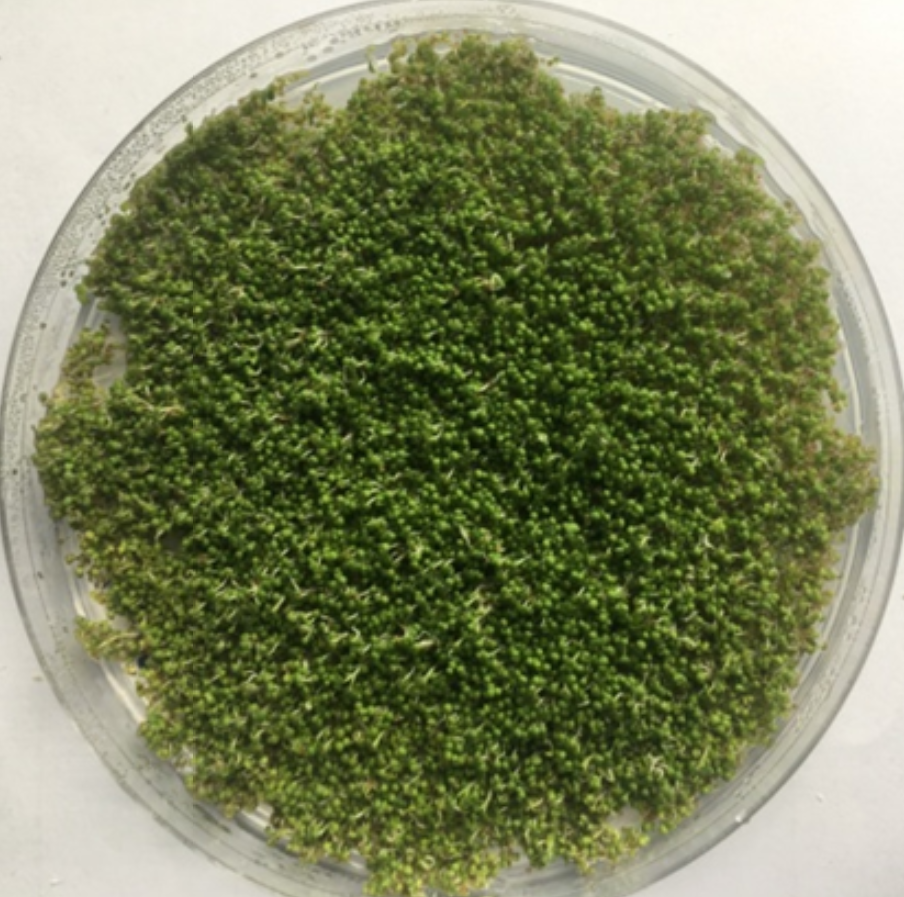


g: OPR4-OE

(A) Hygromycin screening of transgenic *A. thaliana*;


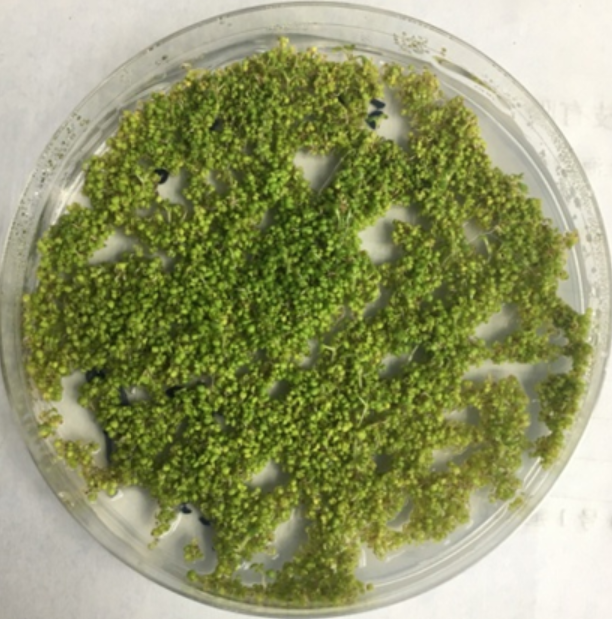


h: OPR4i

(A) Hygromycin screening of transgenic *A. thaliana*.


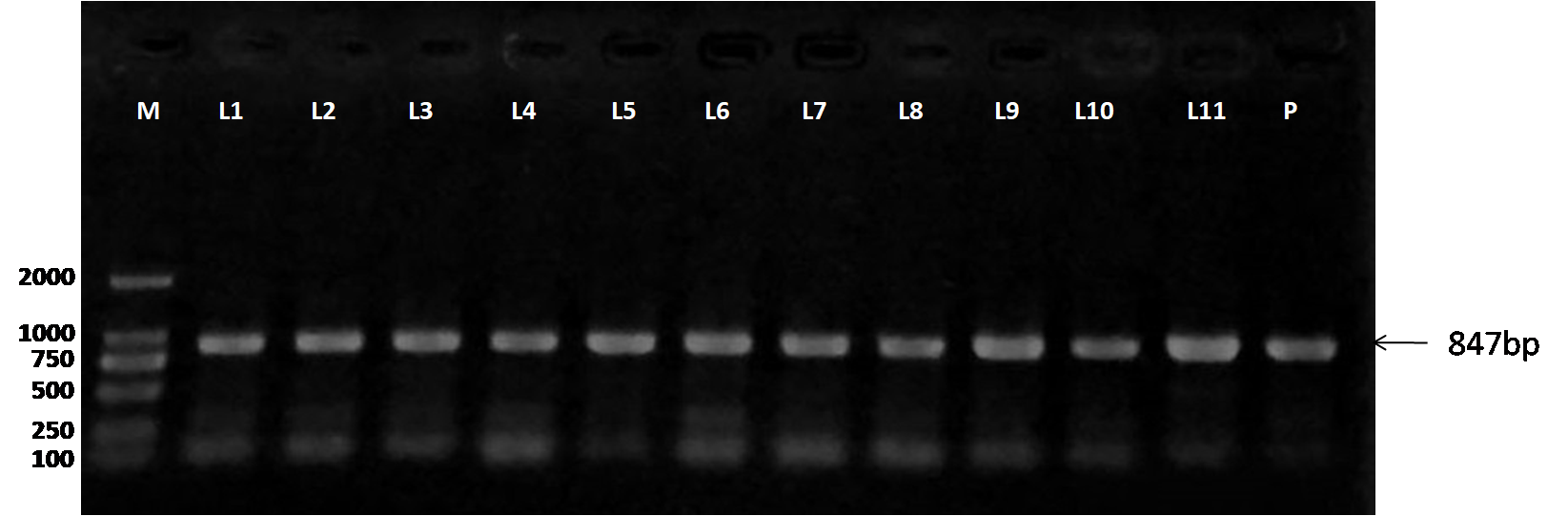


a: pCAMBIA1300-35s-*OPR1*

(B) Identification of hygromycin in transgenic *A. thaliana* (M: DL 2000 bp; P: pCAMBIA1300; a/c/e/g: pCAMBIA1300-35s-*OPR1*/*2*/*3*/*4*; b/d/f/h: pCAMBIA1300-RNAi-*OPR1*/*2*/*3*/*4*).


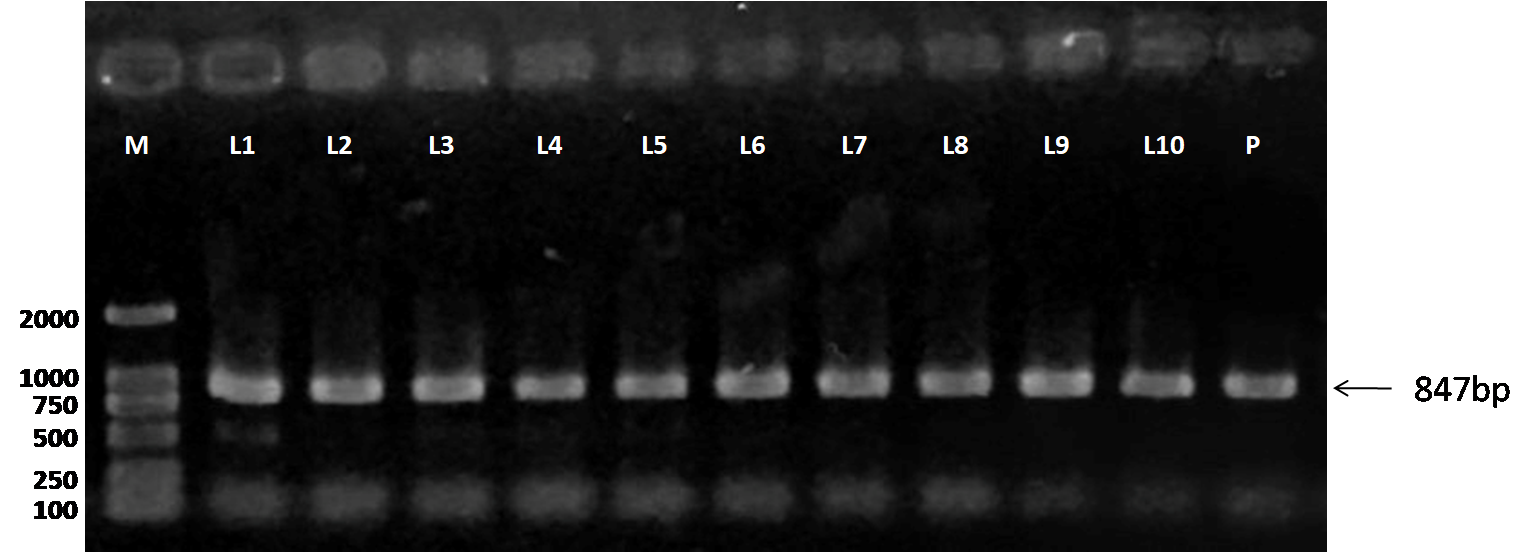


b: pCAMBIA1300-RNAi-*OPR1*

(B) Identification of hygromycin in transgenic *A. thaliana* (M: DL 2000 bp; P: pCAMBIA1300; a/c/e/g: pCAMBIA1300-35s-*OPR1*/*2*/*3*/*4*; b/d/f/h: pCAMBIA1300-RNAi-*OPR1*/*2*/*3*/*4*).


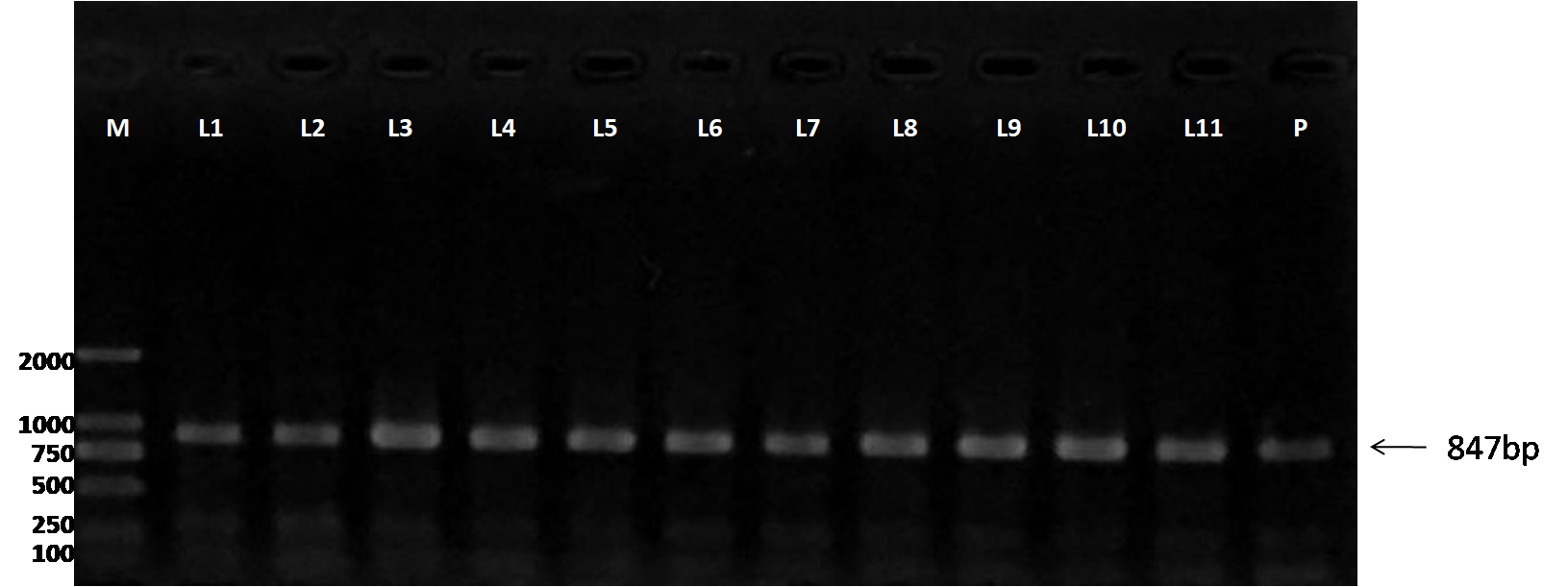


c: pCAMBIA1300-35s-*OPR2*

(B) Identification of hygromycin in transgenic *A. thaliana* (M: DL 2000 bp; P: pCAMBIA1300; a/c/e/g: pCAMBIA1300-35s-*OPR1*/*2*/*3*/*4*; b/d/f/h: pCAMBIA1300-RNAi-*OPR1*/*2*/*3*/*4*).


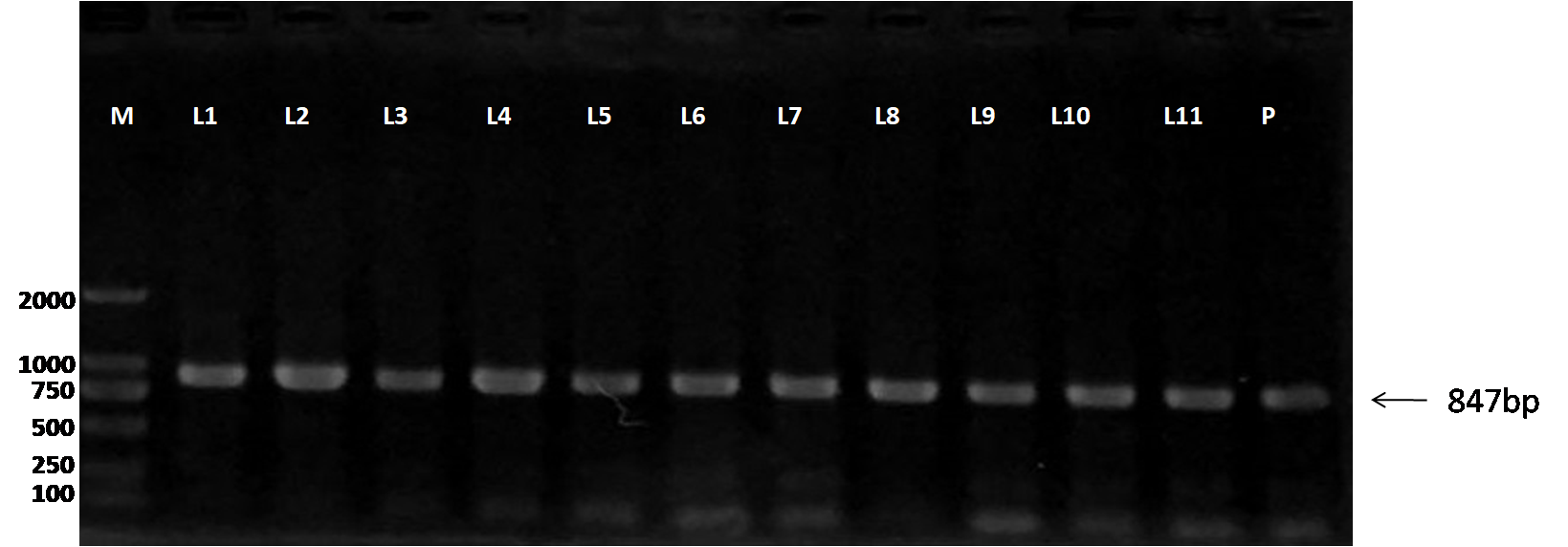


d: pCAMBIA1300-RNAi-*OPR2*

(B) Identification of hygromycin in transgenic *A. thaliana* (M: DL 2000 bp; P: pCAMBIA1300; a/c/e/g: pCAMBIA1300-35s-*OPR1*/*2*/*3*/*4*; b/d/f/h: pCAMBIA1300-RNAi-*OPR1*/*2*/*3*/*4*).


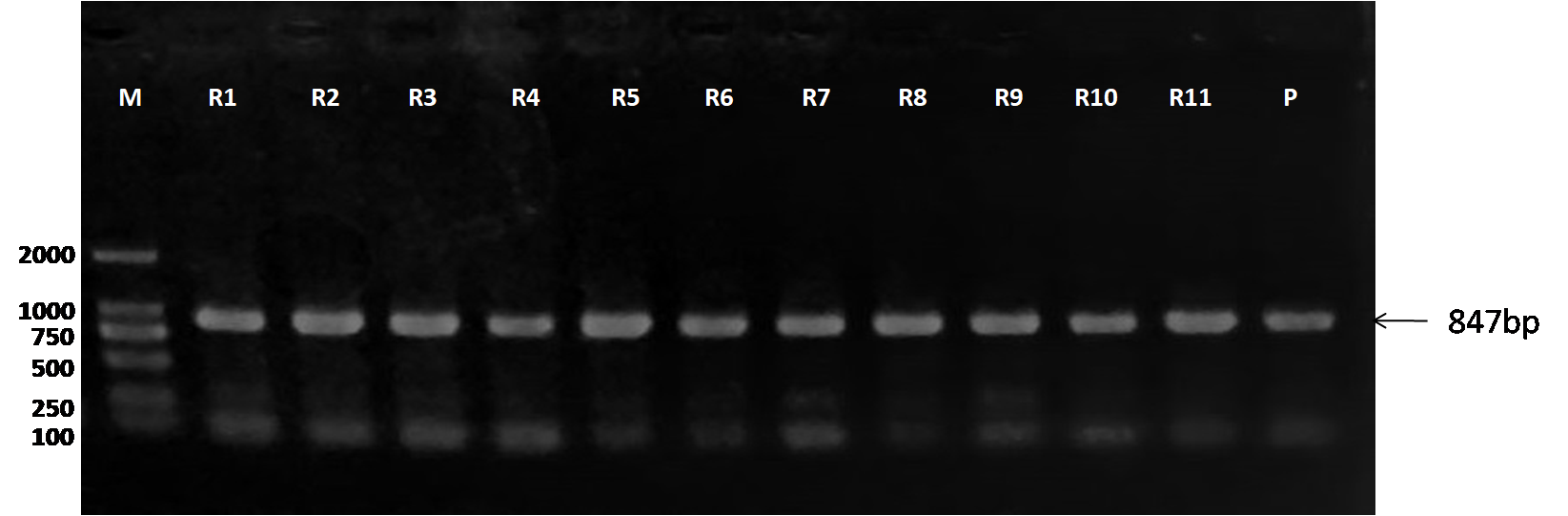


e: pCAMBIA1300-35s-*OPR3*

(B) Identification of hygromycin in transgenic *A. thaliana* (M: DL 2000 bp; P: pCAMBIA1300; a/c/e/g: pCAMBIA1300-35s-*OPR1*/*2*/*3*/*4*; b/d/f/h: pCAMBIA1300-RNAi-*OPR1*/*2*/*3*/*4*).


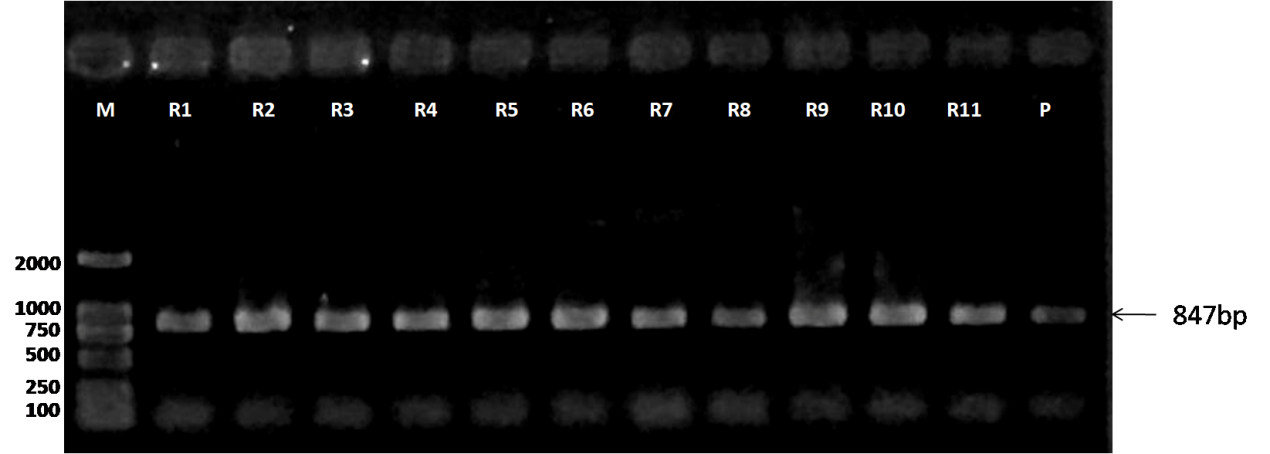


f: pCAMBIA1300-RNAi-*OPR3*

(B) Identification of hygromycin in transgenic *A. thaliana* (M: DL 2000 bp; P: pCAMBIA1300; a/c/e/g: pCAMBIA1300-35s-*OPR1*/*2*/*3*/*4*; b/d/f/h: pCAMBIA1300-RNAi-*OPR1*/*2*/*3*/*4*).


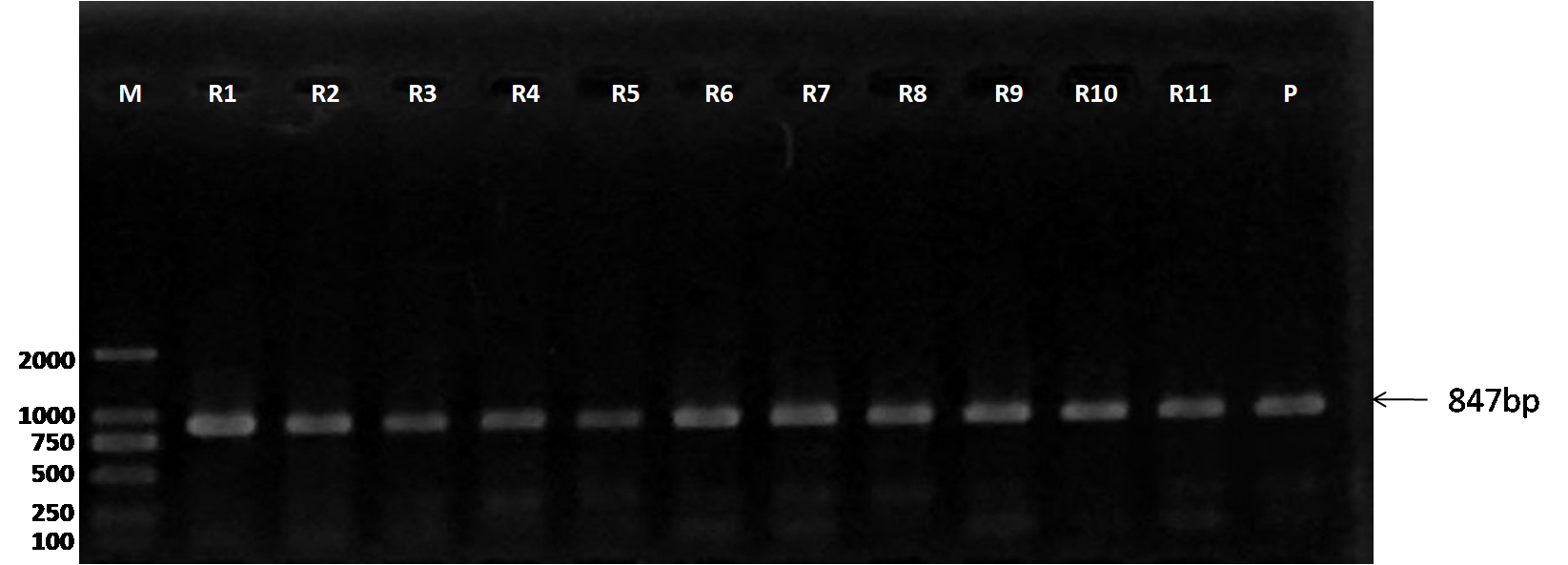
. g: pCAMBIA1300-35s-*OPR4*

(B) Identification of hygromycin in transgenic *A. thaliana* (M: DL 2000 bp; P: pCAMBIA1300; a/c/e/g: pCAMBIA1300-35s-*OPR1*/*2*/*3*/*4*; b/d/f/h: pCAMBIA1300-RNAi-*OPR1*/*2*/*3*/*4*).


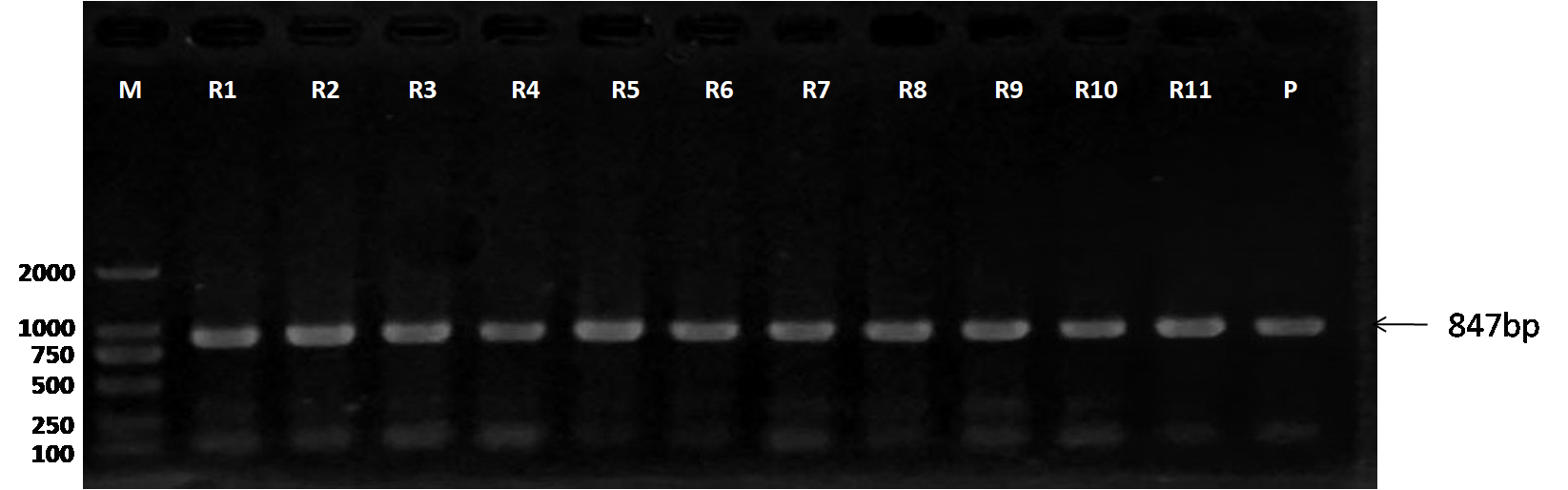


h: pCAMBIA1300-RNAi-*OPR4*

(B) Identification of hygromycin in transgenic *A. thaliana* (M: DL 2000 bp; P: pCAMBIA1300; a/c/e/g: pCAMBIA1300-35s-*OPR1*/*2*/*3*/*4*; b/d/f/h: pCAMBIA1300-RNAi-*OPR1*/*2*/*3*/*4*).

**Figure 6** *Bna00592* KEGG patway network:


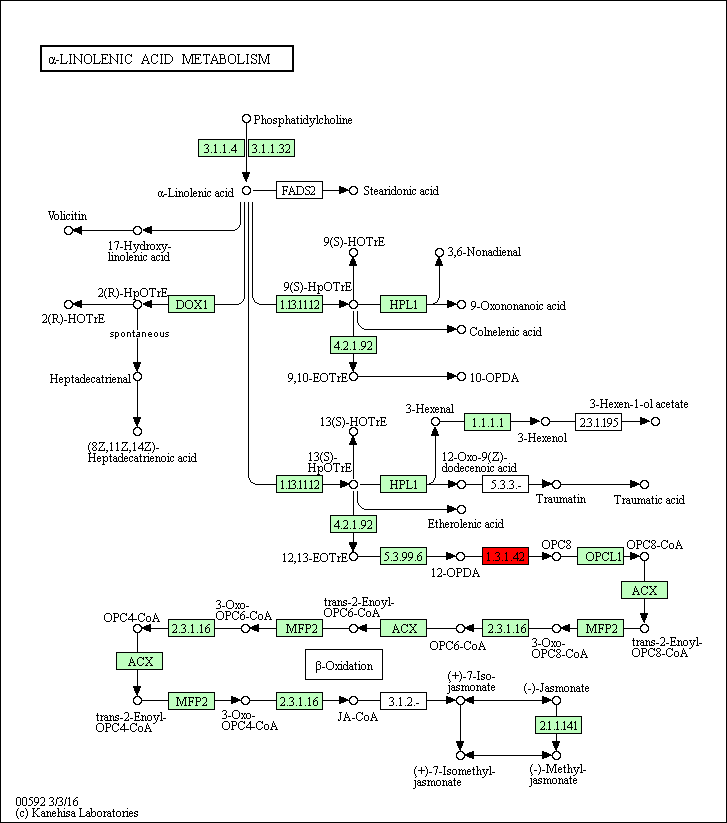

Supplement: Supplementary file 1 — Additional file 1. [file 12870_2022_3549_MOESM1_ESM.docx]
